# Composition of cardiac-derived extracellular vesicles changes with vesicle origin and determines uptake

**DOI:** 10.3389/fcvm.2025.1565104

**Published:** 2025-09-15

**Authors:** Sruti Bheri, Jessica R. Hoffman, Hyun-Ji Park, Swarnima Roychowdhury, Felipe Takaesu, Samuel G. Moore, David A. Gaul, Michael E. Davis

**Affiliations:** ^1^ Wallace H. Coulter Department of Biomedical Engineering, Georgia Institute of Technology & Emory University, Atlanta, GA, United States; ^2^ Molecular & Systems Pharmacology Graduate Training Program, Graduate Division of Biological & Biomedical Sciences, Laney Graduate School, Emory University, Atlanta, GA, United States; ^3^ Department of Molecular Science and Technology, Ajou University, Suwon, Republic of Korea; ^4^ Biochemistry, Cell, and Developmental Biology Graduate Training Program, Graduate Division of Biological & Biomedical Sciences, Laney Graduate School, Emory University, Atlanta, GA, United States; ^5^ Petit Institute of Bioengineering and Bioscience, Georgia Institute of Technology, Atlanta, GA, United States; ^6^ School of Chemistry and Biochemistry, Georgia Institute of Technology, Atlanta, GA, United States; ^7^ Children’s Heart Research & Outcomes (HeRO) Center, Children’s Healthcare of Atlanta & Emory University, Atlanta, GA, United States

**Keywords:** extracellular vesicles (EVs), uptake, proteomics, bioinformatics, lipidomics, stem cell

## Abstract

**Introduction:**

Cardiovascular disease (CVD) is a leading cause of mortality worldwide. The potency of cell-based therapies for CVD is increasingly attributed to the release of small extracellular vesicles (sEVs) which consist of a lipid/protein membrane and encapsulate nucleic cargo. Specifically, sEVs from ckit + progenitor cells (CPCs) and mesenchymal stromal cells (MSCs) are shown to be pro-reparative, with clinical trials conducted. Despite copious research into sEV cargo, the role of parent cell type on sEV membrane composition and its effects on sEV uptake mechanism by recipient cells remain unclear. This is crucial for designing sEV-based therapeutics as uptake mechanism dictates the functionality of the cargo.

**Methods:**

In this study we investigate the role of sEV parent cell and membrane composition on the mechanism of EV uptake by recipient cells.

**Results:**

We find that sEV membrane lipid and protein composition varies by parent cell type. Further, vesicle uptake mechanism varies by both sEV parent cell type and recipient cell type, with clathrin-mediated uptake being the most variable across parent cell conditions. Using a partial least squares regression model, we observe that proteins important in clathrin-mediated uptake (e.g., TPM1, MRC2, FSTL1, LTBP1) are dissimilar to other vesicle uptake mechanisms.

**Discussion:**

This work underscores the importance of the sEV source and membrane composition on uptake, and in turn the importance of selecting specific sEVs based on the target recipient cells for CVD therapies.

## Graphical Abstract

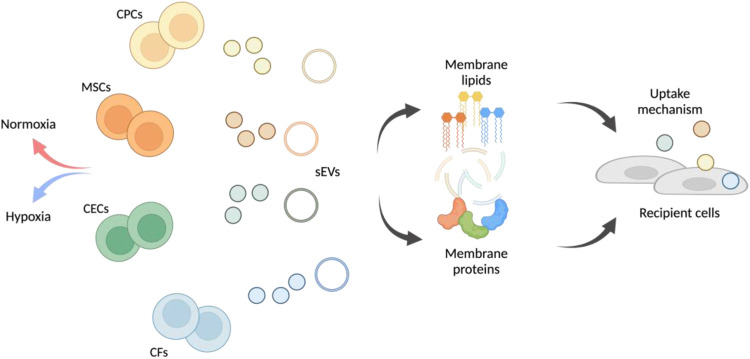

## Introduction

Cardiovascular disease (CVD) is the leading cause of mortality resulting in nearly 1 in every 4 deaths in the United States (1). Cell therapies have been a promising avenue for cardiac repair and recovery. Specifically, cardiac-derived ckit + progenitor cells (CPCs) and mesenchymal stem, or stromal, cells (MSCs) have been potent after CVD, with several clinical trials conducted (2–4). However, a more recent paradigm shift has attributed the benefits of cellular therapies to paracrine signaling, specifically through the release of small extracellular vesicles (sEVs) (5). sEVs are approximately 30–150 nm vesicles released by cells that are composed of an outer lipid/protein bilayer which encapsulates protein and/or nucleic acid cargo.

The sEV's amphiphilic bilayer membrane consists of lipids (e.g., phosphatidylcholine (PC), phosphatidylethanolamine (PE), sphingomyelin (SM), cholesterol (CHOL), ceremide (Cer)), as well as transmembrane proteins [e.g., tetraspanins cluster-of-differentiation-9 (CD9), CD63, CD81] (6, 7). This complex membrane composition is often credited for efficient sEV uptake by cells with minimal clearance (8). Further, the aqueous interior cavity of sEVs contains protein/nuclear cargo (e.g., messenger RNA, microRNA, proteins) which is often enriched from the parent cell, making sEVs potent therapeutics. For example, cardiac-derived sEVs elicit similar cardioprotective responses as the administration of the parent cells, with variations observed based on parent cell type (9).

sEV biogenesis in the parent cells begins in the cytosol with inward budding of the plasma membrane to form the early endosome, transition of the early endosome into the late endosome, secondary inward budding of the late endosome to form sEVs within a multi-vesicular body, and finally fusion of the multivesicular body with the plasma membrane to release the sEVs into the extracellular space (10). Once the sEVs reach the recipient cell, uptake of sEVs can occur through several mechanisms, which are broadly divided into phagocytosis (for particles >1 µm) and pinocytosis (for particles <1 µm) (11). Pinocytosis is common for cells and consists of three major mechanisms: clathrin-mediated uptake, clathrin-independent uptake and macropinocytosis. Clathrin-independent uptake includes caveolae/lipid raft-, RhoA-, flotillin- Arf-6 mediated uptake, surface glycans and CRAF to name a few (12–15). Caveolae-mediated uptake leverages membrane invaginations which are upregulated in sphingolipids and cholesterol whereas RhoA aids with actin-cytoskeletal dynamics. Surface glycans help tune the sEV membrane through either change-based effects of glycan recognition while CRAF activation increase formation of membrane ruffles. Macropinocytosis, a non-specific uptake pathway, leverages such membrane ruffles for non-selective engulfing of extracellular fluid, including sEVs (16–18). All or some of these methods can be utilized by the recipient cell for sEV uptake, after which, sEVs can be trafficked into the cytoplasm or nucleus to initiate functional responses or fuse with the lysosome for degradation.

Although sEV biogenesis and the role of sEV cargo variations in sEV function are well understood, the determining factors relating sEV membrane composition to sEV function, specifically uptake, is still unclear. It has been established that there is asymmetry in the membrane lipids, and this can be enzymatically altered (via flippases, floppases etc.), but the purpose of this structure and ability to alter the lipid membrane is mostly unknown (19). Similarly, it has been reported that variations in sEV surface proteins influence uptake by recipient cells, but this work is limited to cancer-cell derived sEVs and the specific roles of cardiac sEV proteins in uptake are understudied (20). The importance of membrane composition in vesicle uptake is further supported when comparing sEVs to synthetic mimics, which despite similar size and shape, contain less intricate membranes and are often targeted and cleared by the mononuclear phagocyte system (21, 22).

Given that sEVs are derived from their parent cells with inward budding from the cell membrane and then secondary inward budding to form sEVs, the bilayer membrane orientation of sEVs is derived from that of their parent cells. However, it is well established that the sEV membrane does not directly match that of the parent cell, and consists of various proteins (e.g., Annexins, Rab GTPases, TSG101) and lipids (e.g., SMs, Cers) which suggests active loading of these into the sEV membrane (23). Given this, the sEV membrane composition is partially influenced by the parent cell type and there is also sEV-specific protein/lipid incorporation. Therefore, to truly explore the differences in membrane composition on sEV uptake, there remains value in studying sEVs derived from different parent cell soures.

In this study, we explore the relationship between variations in the sEV lipid and protein profiles derived from different parent cell types and elucidate if there is a correlation between these variations and the uptake mechanism employed by the recipient cells. We focus on sEVs isolated from four CVD-relevant cell types, namely, CPCs, MSCs, cardiac endothelial cells (CECs) and cardiac fibroblasts (CFs) to determine sEV membrane-uptake relationships in the cardiac context. We show that sEV uptake mechanism varies both based on the parent and recipient cell with variations in membrane lipid and protein composition by sEV origin. These uptake variations are related to both the parent and recipient cell type, with clathrin-mediated uptake being most distinct.

## Materials and methods

### Isolation and culture of CPCs

Human CPCs were isolated from the right atrial appendage of neonatal pediatric patients undergoing surgical intervention for a congenital heart defect under approval by the Institutional Review Board at Children's Healthcare of Atlanta and Emory University. Neonatal patients were classified as patients 2 weeks of birth at the time of surgery. The CD-117 + cells were separated from the atrial tissue through magnetic bead sorting for c-kit, as previously described (24). CPCs were the cultured in Ham's-F-12 medium (Corning Cellgro®, Corning, NY, USA) with 10% FBS, 1% penicillin-streptomycin, 1% L-glutamine and 0.04% human hFGF-*β*.

### Culture of MSCs, CFs and CECs

BM-MSCs were purchased from Gibco (StemPro™ BM Mesenchymal Stem Cells, Gibco, Waltham, MA) and rat CFs were isolated from adult male Sprague-Dawley rats as previously described (25, 26). Both MSCs and CFS were cultured in Dulbecco's Modified Eagle Medium (DMEM) and Ham's-F-12 medium with 10% FBS, 1% penicillin-streptomycin, 1% L-glutamine and 0.04% hFGF-*β*. Rat primary CECs (R2111, Cell Biologics Inc., Chicago, IL) were cultured in EGM-2 (Endothelial Cell Growth Medium-2 BulletKitTM, Lonza, Bend, OR) supplemented with 1% penicillin- streptomyocin and 2% FBS, 0.4% hFGF-*β*, 0.1% VEGF, 0.1% R3-IGF-1, 0.1% ascorbic acid, 0.1% human hEGF, 0.1% GA-1000, 0.1% heparin, and 0.04% hydrocortisone, as per manufacturer's protocol.

### sEV isolation from CPCs, MSCs, CECs and CFs

CPCs, MSCs, CECs and CFs (∼50 × 10^6^ cells) between passages 8–16 were cultured in 2D until 90% confluency. Cells were then washed 3 times with PBS to remove any serum-media or serum-specific vesicles and quiesced in serum- and growth-factor-free media in normoxic (18% oxygen) or hypoxic (2% oxygen) conditions for 12 h. The oxygen conditioning was performed to recapitulate the ischemic conditions experienced during CVD. Further, previous studies from our group have shown that hypoxic-CPC derived sEVs had more pronounced reparative capacity, suggesting a possible difference in membrane composition (27). sEVs were collected from the conditioned media through differential ultracentrifugation (Optima XPN-100, Beckman Coulter, Indianapolis, IN, USA). Ultracentrifugation was chosen for sEV isolation as it produces a narrow size-range of homogenous sEVs with minimal protein contamination. Briefly, the conditioned media was depleted of cells at 1,000 RPM for 10 min (Centrifuge 5,810 R, Eppendorf, Hamburg, Germany), then depleted of cell debris at 15,000 RPM for 20 min (SW32Ti, Beckman Coulter). Finally, sEVs were pelleted at 31,000 RPM for 114 min (SW41Ti, Beckman Coulter). The bottom 2 ml of each tube containing the sEV pellet was collected and stored at −80 °C for further use.

### sEV characterization

The sEV size and concentration profiles were quantified through NTA (Nanosight NS-300 with NTA 3.4 software, Malvern Panalytical, Malvern, UK) with three 60-second videos per sample. NTA data was captured with an sCMOS camera, Blue 488 laser, 1,300 slide shutter, 512 Gain, 25 FPS, with pure PBS control buffer. Vesicle protein content was assessed with the bicinchoninic acid assay (Pierce™ BCA Protein Assay Kit, Thermo Fisher Scientific, Waltham, MA) as per manufacturer's instructions. Prior to immunoblotting, CPCs, MSCs, CFs, and CECs were lysed using a RIPA Lysis Buffer (Thermo Fischer Science, Waltham, MA) and their protein content was quantified similar to that of vesicle protein content. sEV structure was determined with cryo-electron microscopy (JEOL JEM-1,400, Peabody, MA) with the UltraScan 1,000 CCD to initially visualize the bilayer and henceforth with transmission electron microscopy (JEOL JEM-1400). Finally, vesicle polydispersity index was assessed through DLS (DynaPro Plate Reader III, Wyatt, Santa Barbara, CA).

### Immunoblotting

After cell and vesicle protein content was assessed with BCA, 30 µg of sEVs or cell lysate were mixed in a 1:4 ratio with NuPAGE 4X LDS Sample Buffer (Life Technologies, Carlsbad, CA) and loaded into a 4%–20% Mini-PROTEAN TGX™ Precast Protein Gel (Bio-Rad, Hercules, CA). Samples were run for 45 min at 150 V and then transferred onto a nitrocellulose membrane using a Trans-Blot Turbo Transfer System (Bio-Rad, Hercules, CA) for seven minutes at 25V-2.5A. After the transfer, the membrane was blocked using 1% BSA solution followed by a primary antibody (1:2,000) incubation for 1 h at room temperature. Primary antibodies used were the following: Mouse anti-CD81 (Cat. #10630D, Invitrogen, Waltham, MA), rabbit anti-CD63 (Cat. #52090S, Cell Signaling Technologies, Danvers, MA), mouse anti-ANXA2 (Cat. #03–440-0, Fisher Scientific, Hampton, NH), rabbit anti-BiP (Cat. #3177 T, Cell Signaling Technologies, Danvers, MA), and rabbit anti-GM130 (Cat. #12480S, Cell Signaling Technologies, Danvers, MA). Membranes were then washed three times with PBS-T and were incubated with secondary antibodies (1:2,000) for one hour at 4 °C. Secondary antibodies used were the following: Goat anti-mouse (Cat. #7076S, Cell Signaling Technologies, Danvers, MA) and Goat anti-rabbit (Cat. #7074S, Cell Signaling Technologies, Danvers, MA). Following three PBS-T washes, membranes were incubated with a Super Western™ ECL Substrate (BPS Bioscience, San Diego, CA) and imaged using a ChemiDoc (Bio-Rad, Hercules, CA).

### sEV uptake inhibition

CECs and CFs were cultured until 80% confluency and then seeded at 0.3 × 10^6^ cells/well into 6 well plates. After incubation for cell attachment, the CECs and CFs were quiesced overnight in endothelial bare medium (FBS and growth factor free) or Dulbecco's modified eagle's medium (FBS free), respectively, with 1% penicillin-streptomycin. CECs or CFs were then treated with one of three small molecule inhibitors of sEV uptake: Pitstop-2 (clathrin inhibitor), Nystatin (caveolae/lipid raft mediated uptake inhibitor) or Amiloride (Na^+^/H^+^ pump mediated macropinocytosis inhibitor) for 1 h at 37 °C as per Table 1. Following inhibition, cells were treated with either normoxic or hypoxic calcein + sEVs from CPCs, MSCs, CECs or CFs at 20 µg/ml and incubated at 37 °C for 2 h. The sEVs were pre-stained with membrane dye, calcein (Thermo Fisher Scientific, Waltham, MA) and excess unbound calcein was removed through dialysis overnight at 4 °C (Float-A-lyzer dialysis device, Repligen, Waltham, MA). Calcein + sEVs and calcein-only PBS (no sEVs) were also administered to CECs and CFs (without prior inhibitor treatment) and incubated at 37 °C as negative control, at 4 °C to inhibit sEV uptake as positive control, and with calcein-only PBS to confirm success of dialysis step. After the 2-hour incubation, uptake of sEVs by CECs and CFs was quantified by detection of calcein through flow cytometry. Briefly, CECs or CFs were washed 3 times with sterile PBS to remove free or partially surface-bound sEVs. Cells were then detached and each sample resuspended in 200 µl flow buffer (2% FBS in PBS). Uptake of sEVs was quantified for *λ*_Ex_/*λ*_Em_ = 495/515 nm corresponding to calcein + sEVs. Gating strategy for flow cytometry is detailed in Supplementary Figure S3. Controls for flow cytometry included cells-only (no sEVs), cells + calcein-only PBS, cells + sEV (37 °C, no inhibitor) as negative control and cells + sEV (4 °C, no inhibitor) as positive control.

**Table 1:** Dosage of small molecule inhibitors for sEV uptake.

Small molecule inhibitor	Target uptake pathway	Dosage
Pitstop-2	Clathrin-mediated	10 µM (27)
Nystatin	Caveolae/lipid raft-mediated	50 µg/ml (28)
Amiloride	Na^+^/H^+^ pump mediated macropinocytosis	20 µM (29)

To confirm successful inhibition with the small molecule inhibitors, inhibition of fluorescein isothiocyanate (FITC)-conjugated albumin (A9771, Sigma Aldrich) and tetramethylrhodamine (TRITC)-conjugated transferrin (009-0034, Rockland Immunochemical Inc., Baltimore, MD) were assessed as positive controls. Albumin leverages clathrin-mediated uptake, so was used to assess Pitstop 2 potency. Transferrin can leverage both caveolae/lipid raft-mediated uptake and to a lesser extent, macropinocytosis, so was used to assess Nystatin and Amiloride potency. To quantify potency of each inhibitor, similar to above, CECs and CFs were treated with the small molecule inhibitors (Table 1) for 1 h followed by treatment of 20 µg/ml of albumin-FITC + or transferrin-TRITC for 2 h and uptake of the albumin or transferrin was assessed through flow cytometry for *λ*_Ex_/*λ*_Em_ = 495/515 nm and *λ*_Ex_/*λ*_Em_ = 550/570 nm, respectively.

To validate the protein targets identified from the PLS model, protein-inhibition studies were conducted. Calcein-stained MSC sEVs and CPC sEVs (same methods as above) were incubated with FSTL1 (ThermoFisher Scientific) for CF uptake, MRC2 (ThermoFisher Scientific) for CEC uptake and Cardiac Troponin T (ThermoFisher Scientific) as control in a 1:200 dilution with 2% BSA at room temperature for 1 h. The antibody-sEV solution was spun down in 100,000 kDa filters at 3,000  ×  g for 15 min. The calcein + sEVs with and without antibody-conjugation were treated to the CFs and CECs as above and uptake was assessed through flow cytometry for *λ*_Ex_/*λ*_Em_ = 495/515 nm.

### Live-dead assay

To determine any cytotoxic effects of the small molecule inhibitors on CECs or CFs, cell viability after treatment was assessed. Here, cells were cultured until 80% confluency and seeded at 0.05 × 10^6^ cells/well into 24 well plates. Cells were then treated with the four small molecule inhibitors of uptake (clathrin, nystatin or amiloride) as per Table 1. and incubated for 3 h at 37 °C to recapitulate the sEV uptake inhibition study (5.2.6). Untreated CECs and CFs at 37 °C and 4 °C were positive and negative controls, respectively. After 3 h, cells were detached and treated with Zombie Red™ viability dye (423109, Biolegend, San Diego, CA) as per manufacturer's instructions and incubated at room temperature for 15–30 min. Cells were then washed and resuspended in flow buffer (2% FBS in PBS). Percentage of Zombie Red + cells were quantified by flow cytometry for *λ*_Ex_/*λ*_Em_ = 561/624 nm.

### Mass spectrometry—proteomics

#### Sample prep

sEV samples were prepped with a slightly modified EasyPep (Thermo Fisher Scientific) digestion protocol. Briefly, 100 µl of each sEV-enriched sample was diluted with 200 µl of EasyPep lysis buffer. Samples were then reduced and alkylated with 10 mM TCEP and 40 mM CAA at 95 °C for 10 min. The samples were bath sonicated for 10 min and allowed to cool to room temperature. Samples were then digested overnight with a 1:1 endoproteinase LysC:trypsin mixture (2 µg of each enzyme was used). Samples were then desalted with EasyPep cleanup columns following manufacturer's protocol and then dried down with a SpeedVac vacuum concentrator.

#### LC Ms/Ms

All samples were analyzed on the Evosep One (Evosep, Odense, Denmark) system using an in-house 15 cm, 150 mm I.D. capillary column packed with 1.9 µm Reprosil-Pur C18 beads (Dr. Maisch, Ammerbuch, Germany) using the pre-programmed 88-minute gradient, at a frequency of 15 samples per day. Mass spectrometry was performed with a Orbitrap Q-Exactive Plus (Thermo Fisher Scientific) in positive ion mode using data-dependent acquisition with a top 20 method. Each cycle consisted of one full MS scan followed by as many as 20 MS/MS scans. MS scans were collected at a resolution of 70,000 (400–1,600 m/z range, 3 × 10^6^ AGC, 100 milliseconds maximum ion injection time). All higher energy collision-induced dissociation (HCD) MS/MS spectra were acquired at a resolution of 17,500 (1.6 m/z isolation width, 28% collision energy, 1 × 10^5^ AGC target, 100 milliseconds maximum ion injection time). Dynamic exclusion was set to exclude previously sequenced peaks for 30 s within a 10-ppm isolation window.

#### Database searching and protein quantification

All raw files were searched using Thermo's Proteome Discoverer suite (version 2.4.1.15) with Sequest HT. The spectra were searched against either a human (78,860 target sequences) or rat Uniprot database (29,918 target sequences) both downloaded in May 2022. Search parameters included 10 ppm precursor mass window, 0.05 Da product mass window, dynamic modifications methionine (+15.995 Da), deamidated asparagine and glutamine (+0.984 Da), phosphorylated serine, threonine, and tyrosine (+79.966 Da), and static modifications for carbamidomethyl cysteines (+57.021 Da). Percolator was used to filter PSMs to 0.1%. Peptides were grouped using strict parsimony and only razor and unique peptides were used for protein level quantitation. The Minora plugin was used to perform the label-free quantification. Only unique and razor (i.e., parsimonious) peptides were considered for quantification. All raw files and search outputs are available at https://www.synapse.org/#!Synapse:syn30941609.

#### Mass spectrometry—lipidomics

All solvents were LC-MS grade and were purchased from ThermoFisher Scientific. All stable isotope-labeled internal standards (IS) were purchased from Avanti Polar Lipids (Alabaster, Alabama): of PC [15:0–18:1(d7)]; PE [15:0–18:1(d7)]; PS [15:0–18:1(d7)]; PG [15:0–18:1(d7)]; PI [15:0–18:1(d7)]; LPC [18:1(d7)]; LPE [18:1(d7)]; Chol Ester [18:1(d7)]; DG[15:0–18:1(d7)]; TG [15:0–18:1(d7)-15:0]; SM [18:1(d9)]; and cholesterol (d7). IS were added to the extraction solvent at a final concentration in the 0.1–8 μg/ml range.

Samples were stored at −80 ⁰C until extraction. Lipids were extracted from exosome samples with 400 µl of iso-propanol (IPA). The samples were vortex mixed for 2 min at 3,000 rpm followed by sonication for 5 min. The samples were dried with a CentriVap (Labconco), reconstituted 50 µl IPA, vortexed mixed, sonicated for 5 min, and centrifuged at 21,100xg for 5 min. Supernatant was transferred to an LC Vial and stored at 4 °C until analysis. Sample blanks were prepared using PBS, serum-free Ham's-F-12, serum-free DMEM, and serum-free EGM-2. An aliquot from each supernatant was combined to create a pooled sample used as a quality control (QC).

Lipid LC-MS data were acquired using a Vanquish (ThermoFisher Scientific) chromatograph fitted with a ThermoFisher Scientific Accucore™ C30 column (2.1 × 150 mm, 2.6 µm particle size), coupled to a high-resolution accurate mass Orbitrap ID-X mass spectrometer (ThermoFisher Scientific) for both positive and negative ionization modes. The mobile phases were 40:60 water:acetonitrile with 10 mM ammonium formate and 0.1% formic acid (mobile phase A) and 10:90 acetonitrile:isopropyl alcohol, with 10 mM ammonium formate and 0.1% formic acid (mobile phase B). The chromatographic method used the following gradient program: 0 min 80% A; 1 min 40% A; 5 min 30% A; 5.5 min 15% A; 8 min 10% A; held 8.2 min to 10.5 min 0% A; 10.7 min 80% A; and held until 12 min. The flow rate was set at 0.40 ml/min. The column temperature was set to 50 °C, and the injection volume was 5 µl. For analysis the electrospray ionization source was operated at a vaporizer temperature of 320 °C, a spray voltage of 3.5 kV for positive ionization mode and 2.5 kV for negative ionization mode, sheath, auxiliary, and sweep gas flows of 40, 8, and 1 (arbitrary units), respectively, and capillary temperature of 275 °C. The instrument acquired full MS data with 240,000 resolution over the 150–2000 *m/z* range. Samples were analyzed in random order with pooled QC injections collected at minimum every tenth injection. LC-MS/MS experiments were acquired using a data dependent acquisition (DDA) strategy to aid in compound identification. MS^2^ survey spectra were collected with a resolution of 60,000. Precursors were isolated with a 0.8 m/z window and activated with stepped 15, 30, 45% HCD and 35% CID energies. HCD activated product ions were measured in the orbitrap at 30,000 resolution and CID activated product ions were measured in the ion trap. Dynamic exclusion was set at 5 s and ions with charges greater than 2 were omitted.

Data were processed with Compound Discoverer V3.0 (ThermoFisher Scientific) to yield an aligned feature table containing *m/z*, RT, and relative peak areas. Detected features were filtered with background and QC filters. Features with abundance lower than 5x the background signal in the sample blanks and that were not present in at least 50% of the QC pooled injections with a coefficient of variance (CV) lower than 30% were removed from the dataset. The pooled QC injections were used to drift correct each individual feature.

Lipid annotations were accomplished based on accurate mass and relative isotopic abundances (to assign elemental formula), retention time (to assign lipid class), and MS^2^ fragmentation pattern matching to local spectral databases built from curated experimental data. When possible, features were matched to authentic compounds were identified with MSI level 1. Features that were matched to local databases were annotated with MSI level 2 or compound-class annotations with MSI level 3. Unknown features were assigned MSI level 4. Lipid annotations are highly subject to the available structural information to assign alkyl chain lengths, alkyl chain position, double bond position, and double bond stereochemistry. Annotations reflect the available structural information, which results in a feature with multiple possible lipid structures.

### Omics data processing

Label-free quantification lipid and protein mass spectrometry experiments were performed. Peak intensities for protein and lipid data were considered. Features were annotated and proteins/lipids with medium or high confidence were considered. For duplicate lipid names, lipids with the lowest average coefficient of variation value among quality-control samples were considered. Lipid isomer peaks (identical annotations with different retention times, Δ > 0.3 min) were summed. Data from all sEVs and oxygen conditions (*n* = 24) were combined for proteins and lipids.

Lipids were filtered to first remove features with more than 12 missing values. Missing values were replaced by ⅕ the minimum value in the corresponding sample. Data were log10 transformed and scaled. Proteomics data were filtered to retain proteins with at least 2 PSM in a sample and remove proteins with more than 12 missing values. Missing values were replaced by ⅕ the minimum value in the corresponding sample. Data were log10 transformed and scaled.

Principal component analyses of normalized lipidomic and proteomic data were performed using R built-in prcomp function. Differential abundance analyses of proteins from CPCs, MSCs, CECs, and CFs was performed after removing data points > mean + 1.5*IQR or < mean – 1.5*IQR using a two-sided, unpaired *t*-test. Proteins with > 2-fold difference in differential abundance analyses were included in pathway analysis using enrichR.

### Partial least squares regression model

To connect lipid/protein omics data to EV uptake mechanism, a partial least squares (PLS) regression model was constructed using normoxic EV data. Lipid and protein data were log_10_ transformed, mean-centered, and scaled. Experimental uptake data were scaled and centered with the mdatools package in R before use in regression models. The mdatools package was also used to construct PLS regression models using the SIMPLS algorithm. First, 3-component models were constructed using all features and leave-one-out cross validation. VIP scores were calculated for the model and lipids/proteins with an average score >1 across all uptake mechanisms were selected. Then, a 2-component final, reduced model was constructed from the VIP lipids/proteins. Model performance of the cross-validated training set was assessed with root-mean-square error (RMSE) and R^2^ measurements. Finally, the model (constructed from normoxic sEV data) was tested on the hypoxic sEV data set.

### Code availability

All code and protein/lipid data are available via github.com/jhoff18/sEV_Uptake_2024/.

### Transmission electron microscopy and cryo-electron microscopy

For negative stain–transmission electron microscopy (NS-TEM), exosome samples were absorbed for 1 min onto glow-discharged, carbon-coated copper grids (Electron Microscopy Sciences, Hatfield, PA, USA). Samples were blotted from the grid, washed on three drops of water, and negatively stained on a drop of freshly prepared and filtered, 2% uranyl acetate solution. Samples were imaged at a nominal magnification of 50Kx using a JEOL JEM-1,400 transmission electron microscope (JEOL, Japan) operating at 80 kV. Electron micrographs were acquired on a 2,048 × 2,048 charge-coupled device (CCD) camera (UltraScan 1,000, Gatan Inc, Pleasanton, CA, USA). For cryo-transmission electron microscopy (cryo-TEM), samples were applied to glow-discharged, 200 mesh, Lacey grids, and plunge frozen in liquid ethane using a Vitrobot Mark IV (ThermoFisher, Hillsboro, OR). Cryo-TEM images were acquired on the JEOL JEM-1400 with the UltraScan 1,000 CCD at a nominal magnification of 30Kx.

### Tube formation assay

EV blocking was done using a 1:100 dilution antibody treatment into each EV sample followed by overnight spinning at 4 °C. Cardiac endothelial cells (CECs) were cultured to 80% confluency and quiesced with EBM-2 Basal Medium (Lonza, Basel, Switzerland) overnight. The following day an IBIDI µ-slide (IBIDI, Fitchburg, WI) was coated with Geltrex (Gibco, Waltham, MA) and incubated at 37 °C for 1 h. Quiesced CECs were seeded into each well at a 20,000 cells/well density and treated with 3 µgs of CPC- or MSC-EVs. CECs were then placed in a 37 °C incubator for 6 h. IgG, and CD81 antibodies were used as a negative blocking control, while EGM-2 Endothelial Cell Growth Medium (Lonza, Basel, Switzerland) was used as a positive control. Following their incubation, 10 mM of Calcein-AM (Thermo Fisher Scientific, Waltham, MA) were used to stain CECs. Phase contrast and fluorescent images were taken, and tube parameters were quantified using the Angiogenesis Analyzer imageJ plugin (Fiji, National Institutes of Health, Bethesda, MD).

### Statistical analysis

All statistical analysis was performed using GraphPad PRISM 8 software (GraphPad, San Diego, CA) with specific testing details outlined in figure captions.

## Results

### sEVs successfully isolated and characterized from all four parent cell types

**Figure 1:**
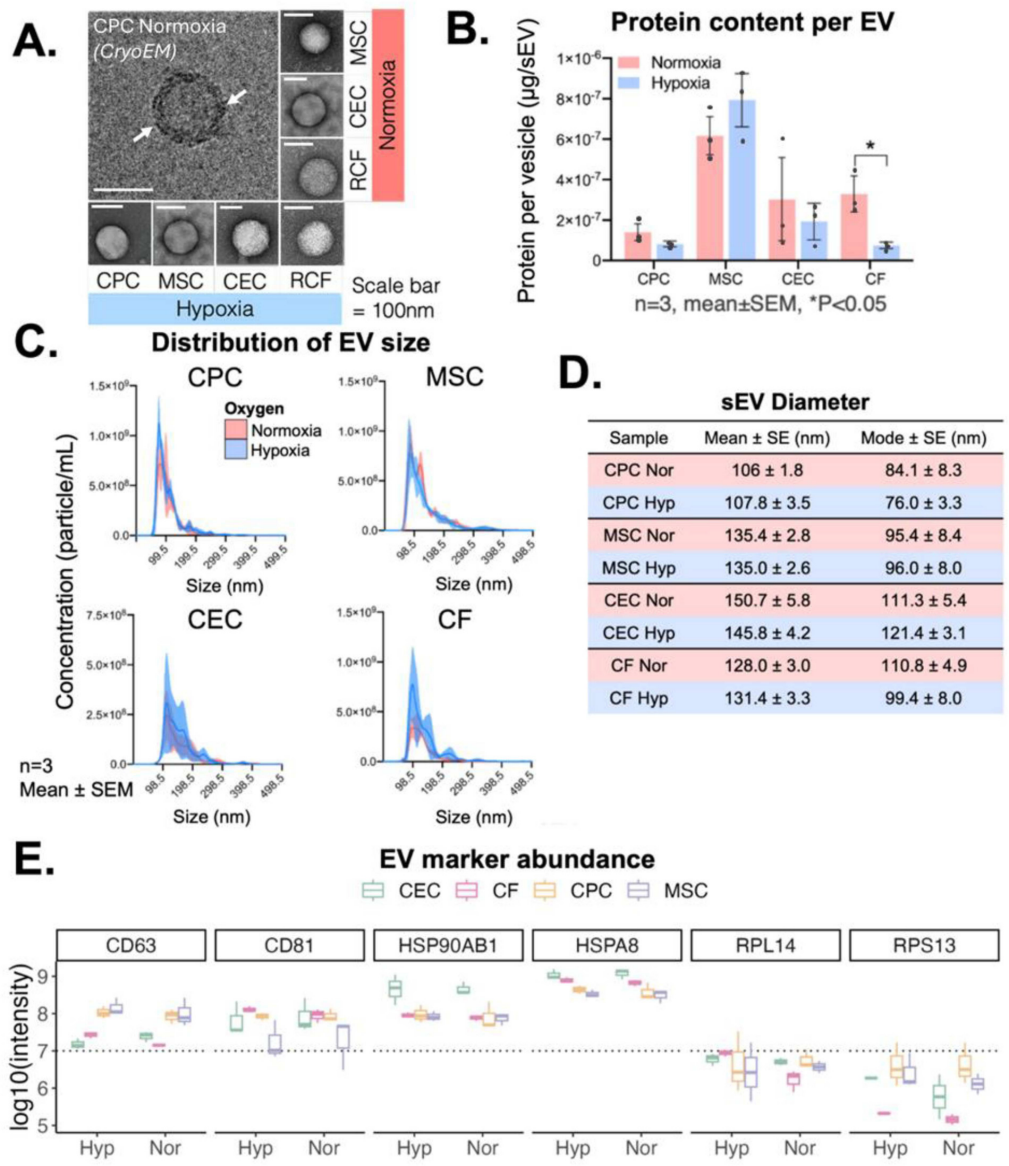
sEV isolation and characterization from CPCs, MSCs, CECs and CFs. **(A)** Cryo- and transmission electron microscopy images of isolated sEVs from all four cell types. **(B)** Variation in protein content across 4 cell types based on oxygen conditioning. **(C)** Concentration-size profile of isolated sEVs with NTA. **(D)** Diameter of sEVs from all four cell types. **(E)** Presence of transmembrane markers (CD63, CD81), cytosolic markers (HSP90AB1, HSPA8), and ribosomal proteins (RPL14, RPS13) measured with mass spectrometry. Scale bar = 100 nm (CryoEM and TEM). *n* = 3 biological replicates, Mean ± SEM, Significance was tested with two-way Student's paired *t*-test. *P < 0.05.

CPCs, MSCs, CECs and CFs were cultured in 2D until 90% confluency and then cells were incubated in serum-free media in normoxic (18% oxygen) or hypoxic (2% oxygen) conditions for 12 h. sEVs were then isolated from the conditioned media through differential ultracentrifugation as described in the methods section. The sEV shape was detected through transmission electron microscopy and the membrane lipid-bilayer (white arrows) with cryo-electron microscopy (Figure 1A). The total protein content of each of the sEVs was assessed with a bicinchoninic acid assay, and most sEV protein content was independent of oxygen conditioning with only CF-sEVs having significantly higher protein per vesicle with normoxic incubation (Figure 1B). The presence of CD63, CD81 and Annexin A2 and the absence of BiP and GM130 for all the sEVs was confirmed with immunoblotting (Supplementary Figure S1). Further, sEVs had similar size-concentration profiles, were isolated at approximately 2 × 10^8^ to 5 × 10^8^ particles/ml and had a mean diameter between 100 and 150 nm (Figures 1C,D and Supplementary Figure S2). Finally, sEV diameter was within the expected range for all samples (106 ± 1.8 to 150.7 ± 5.8 nm) and all sEVs displayed important sEV-markers: CD63, CD81, HSP90 and HSPA8 (Figure 1E).

### sEV uptake mechanism varies based on parent cell type and recipient cell type

To assess the variations in uptake mechanism based on parent cell type, we delivered the sEVs to 2D cultures of CECs and CFs. We chose these cell types as they are abundant in cardiac repair and are important cell types during remodeling and recovery after MI (30). We delivered sEVs from CPCs/MSCs to represent stem and progenitor source sEVs and sEVs from CEC/CF as they are associated with cardiac remodeling and cardioprotection after infarction (31, 32). Three uptake mechanisms, clathrin-mediated, macropinocytosis, and caveolae/lipid-raft-mediated, were studied using small-molecule inhibitors to block each uptake pathway. Calcein + sEVs were then administered and vesicle uptake was assessed using flow cytometry. Concentrations of each inhibitor was determined based on prior literature review (Table 1), and successful inhibition was first confirmed using albumin and transferrin as positive controls (Supplementary Figure S4A). All three small molecular inhibitors reduce albumin/transferrin uptake significantly (<10%). Finally, as small molecular inhibitors may be cytotoxic, the effect of our selected concentrations on CECs and CFs were tested with a viability assay (Supplementary Figure S4B). None of the selected small molecule inhibitor doses significantly affected CEC or CF viability, so these concentrations were used here forth.

**Figure 2:**
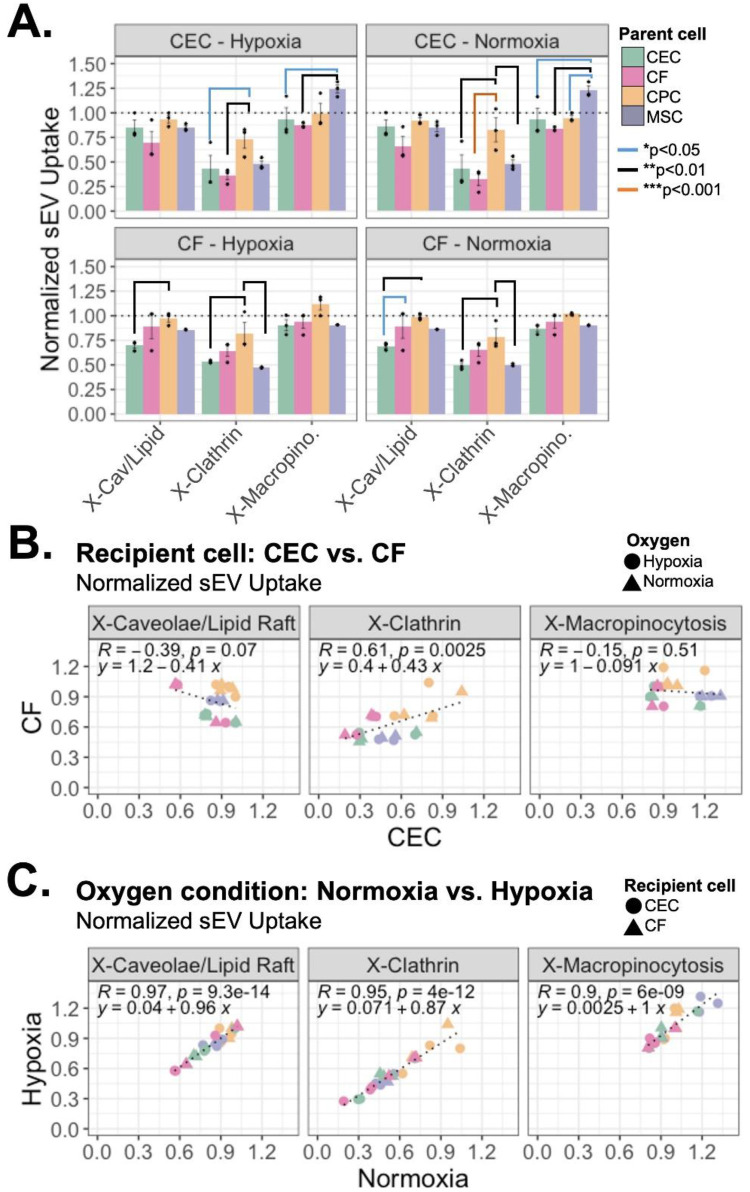
Mechanism of sEV uptake by recipient CECs and CFs. Uptake mechanisms in recipient CEC/CF cells inhibited by small molecule inhibitors clathrin (pitstop-2), macropinocytosis (amilioride), caveolae/lipid-raft (nystatin). **(A)** Uptake of sEVs from the four parent cell types assessed through flow cytometry and normalized to uninhibited controls. *n* = 2-3 biological replicates. Mean ± SEM. Two-way ANOVA with tukey's *post hoc*. Dotted line = uptake of sEV only (without inhibitors). **(B)** Correlation of normalized uptake between CEC and CF recipient cells and **(C)** between normoxia and hypoxia treatments. R = Pearson correlation coefficient.

After confirmation of uptake inhibition and absence of cytotoxicity from the four small molecule inhibitors, we explored the variations in uptake mechanism utilized by the different sEVs through flow cytometry. Of note, the different uptake mechanisms likely occur concurrently, therefore the inhibition of each uptake pathway separately isn't expected to inhibit sEV uptake completely. Differences in uptake mechanisms were observed in both normoxic and hypoxic conditions which varied by both sEV-parent cell type (CPC, MSC, CEC, CF) and the recipient cell type (CEC or CF) (Figure 2A). Administration of sEVs to CECs demonstrated that sEV-parent cell type played a role in clathrin and macropinocytosis based uptake, but not in caveolae/lipid raft mediated uptake. Whereas administration of sEVs to CFs showed more variance across sEV-parent cell types among clathrin and caveolae/lipid raft-mediated mechanisms, but not in macropinocytosis mediated uptake. MSC- and CEC-sEVs had more variability in their uptake extent. Further, MSC-sEV administration to CECs and CPC administration to CFs show almost equal or higher vesicle uptake when macropinocytosis is inhibited. Note that the sEV uptake (normalized to 1) does not represent the “maximum” uptake but rather the “combined effect” of uptake when these three uptake mechanisms are not inhibited. Therefore, it is feasible that inhibiting macropinocytosis could indirectly increase sEV uptake further than uninhibited sEV treatment.

Interestingly, there was not a positive correlation of normalized sEV uptake between recipient cells (CECs and CFs) for micropinocytosis and caveolae/lipid raft-mediated uptake mechanisms (Figure 2B). However, similar patterns of vesicle uptake mechanisms were observed under hypoxic and normoxic conditions (Figure 2C). There was a strong, positive correlation of normalized sEV uptake between normoxic and hypoxic conditions for all uptake mechanisms (R > 0.9). Taken together, this suggests that oxygen conditioning may not play a significant role in sEV uptake, but source and recipient cells affect both uptake mechanism and extent.

### Lipidomics profiling of sEVs shows differences based on parent cell type

**Figure 3:**
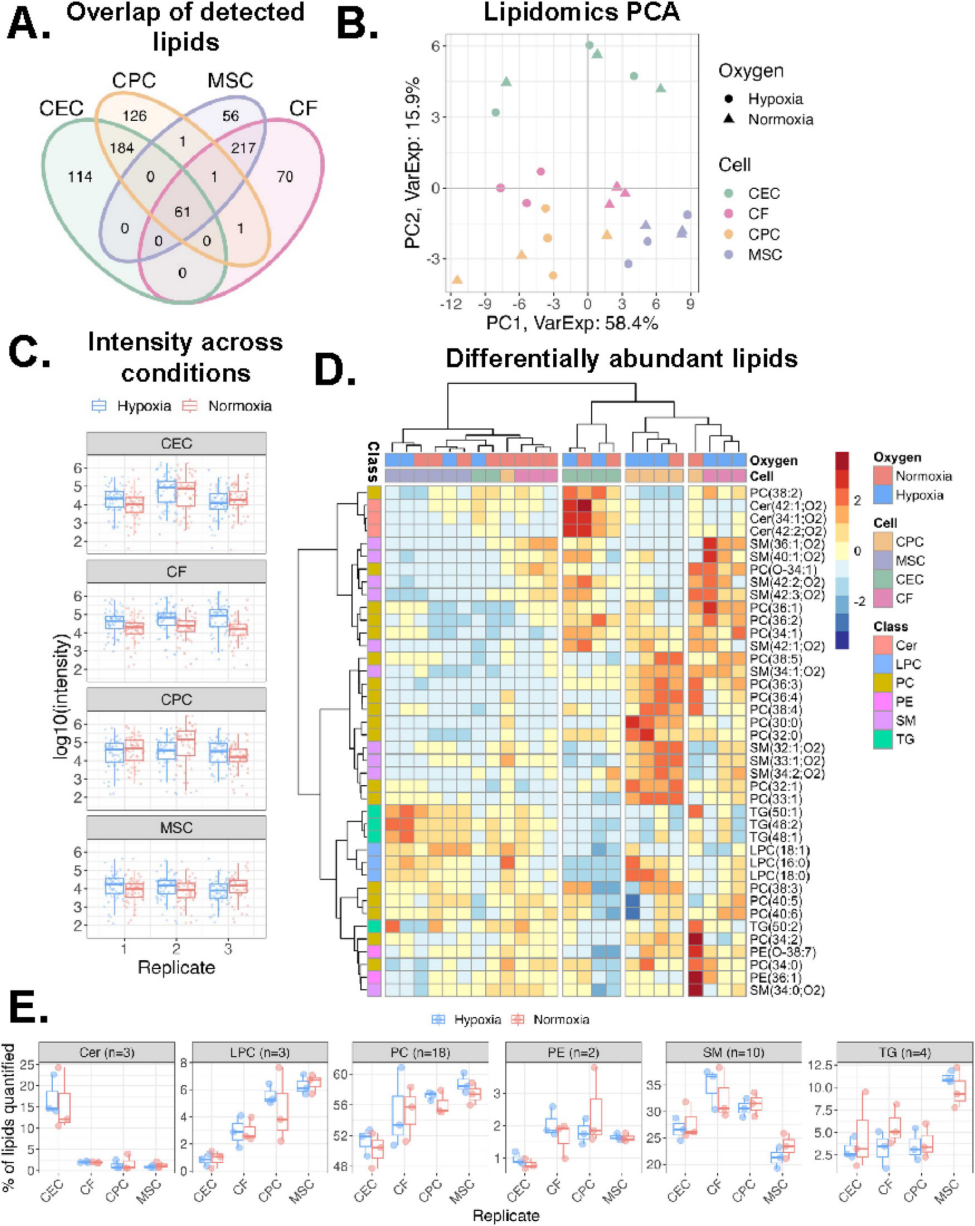
Variations in lipidomics profile of sEVs. **(A)**Venn diagram of lipids detected across all four cell types. **(B)** Principal component analysis of lipidomics data. Samples clustered by sEV parent cell type across component 1 (58% variance) and component 2 (16% variance). **(C)** Log_10_ intensity of lipids detected across sample groups. Middle line and box length represent the median and interquartile range, respectively. Outside “whisker” lines represent minimum and maximum. **(D)** Heatmap of all lipid classes and their abundance across the 4 cell types and oxygen conditions. Values from each sample scaled and mean-centered. Heatmap displays values scaled by row/lipid. Clustering by ward.D, sample dendrogram cut into four clusters. **(E)** Boxplot of percentage of each lipid class within a sample. Lipid (*n* = 40 total) intensities were summed and a percentage of total signal for each sample are reported. *n* = 2-3 biological replicates. PC = phosphatidylcholine, LPC = lysophosphatidylcholine, Cer = ceramide, PE = phosphatidylethanolamine, SM = sphingomyelin, TG = triglyceride.

Next, we explored the membrane of sEVs by conducting mass spectrometry analysis of the sEV lipids and proteins. The lipids detected in all the sEVs varied by parent cell type with CEC and CPC sEVs having the most unique detected lipid features at 114 and 126, respectively (Figure 3A). Principal component analysis revealed that samples cluster by parent cell type, with little influence by oxygen conditioning for all sEVs except CF-derived sEVs (Figure 3B). We next compared lipid intensities across oxygen conditions for all samples and observed similar distributions of lipid abundances (Figure 3C). Next, we looked at differentially abundant lipids by condition and parent cell type (Figure 3D). CPC sEVs are the most upregulated in lipid classes, especially in PC and SM and MSC sEVs are the least upregulated in most lipid classes. Within the MSC lipid profile, the LPC and triglyceride (TG) lipid classes are slightly more abundant, whereas CEC are more abundant in ceramides (Cer). Further, the CF lipid profile is relatively distributed across all the major lipid classes, being the only group with a notable difference by oxygen condition. To further elucidate some of the parent-cell based variability, we also show lipid profiles categorized by class (Figure 3E). Of the quantified lipids, LPC and PE were less prevalent in CECs and SM was less prevalent in MSCs. However, we note that this analysis is limited by the number of quantified lipids per category. Taken together, this underscores that sEV lipid profile is variable and does depend on sEV parent cell type.

### Proteomics profiling of sEVs shows differences based on parent cell type

**Figure 4:**
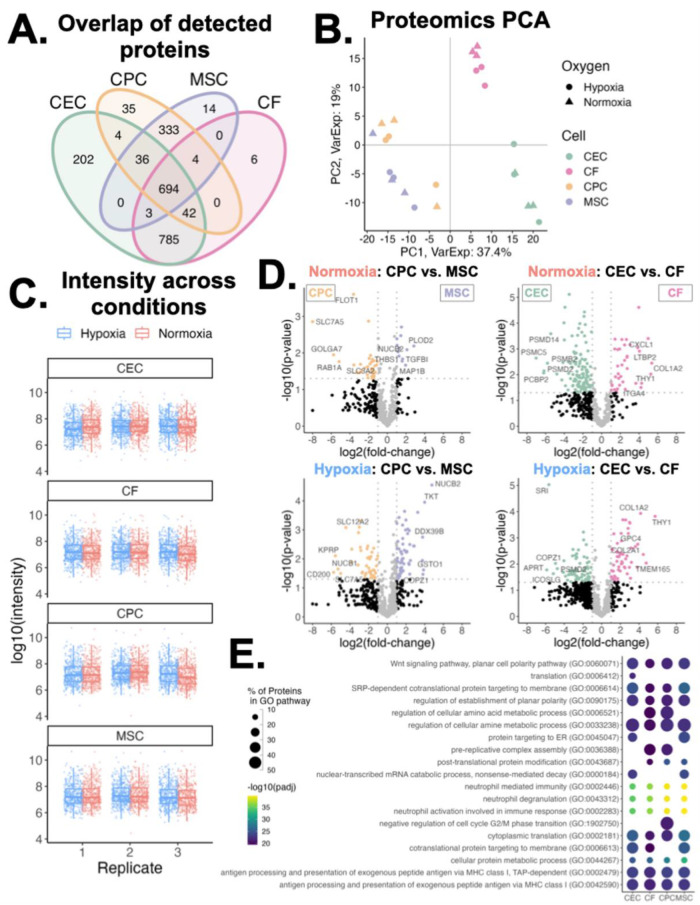
Variations in proteomics profiles of sEVs. **(A)** Venn diagram of proteins detected across all four sEV parent cell types. **(B)** Principal component analysis of proteins clustered across component 1 (37% variance) and component 2 (19% variance), by cell type and oxygen conditioning. **(C)** Log_10_ intensity of proteins detected across sample groups. Middle line and box length represent the median and interquartile range, respectively. Outside “whisker” lines represent minimum and maximum. **(D)** Volcano plots of differentially abundant proteins in normoxic and hypoxic conditioned sEVs (CPC vs. MSC and CEC vs. CF). Top differentially abundant proteins are labeled. Two-sided, unpaired *t*-test. **(E)** Enrichment of GO Biological Process pathways from proteins abundant in normoxic conditioned sEVs. Pairwise comparisons made between each parent cell-type; differentially abundant proteins (fold-change > 2 in any comparison) were used for pathway analysis. *n* = 2-3 biological replicates. Top 15 pathways for each sEV group shown.

We also explored the variations in sEV proteins across the different cell types. Compared to the lipid features, the proteins detected in the sEVs varied far less by parent cell type with CEC and CF sEVs accounting for 3 distinct proteins and CPC and MSC sEVs accounting for another 3 (Figure 4A). Principal component analysis of proteomics data showed a similar result as lipidomics PCA: samples cluster by parent cell type, independent of oxygen conditioning (Figure 4B). Assessing protein signal across conditions we observe consistent distributions of protein intensities across oxygen conditions, cell type, and replicates (Figure 4C). Next, for both normoxic and hypoxic groups, we compared differential protein abundance between CPC and MSC sEVs and CEC and CF sEVs (Figure 4D). Interestingly proteins RAB1A and GOLGA7, involved in transport and FLOT1, a caveolae-associated integral membrane protein were upregulated in CPCs, compared to MSCs. Extracellular matrix-related proteins TGFB1, MAP1B, and THBS1 were upregulated in MSCs, compared to CPCs. When comparing CECs and CFs, several proteasome proteins were upregulated in CECs and chemokine and collagen proteins were upregulated in CFs. Further, we also compared differentially abundant proteins between normoxia and hypoxia for CEC/CF and MSC/CPC (Supplementary Figure S5). This showed that the differential abundance of proteins is similar between the sEV groups when exposed to different oxygen conditions. Given this, we focussed on normoxic proteins henceforth. Looking at the downstream protein pathways of these differentially expressed proteins, we observe that CEC and MSC sEVs are more pronounced in ER targeting proteins while CPC sEVs are distinct in cell cycle phase transition proteins (Figure 4E). Perhaps as expected, all four sEV types show enrichment of membrane proteins involved in protein targeting to membrane and cytoplasmic translation, which are important in vesicle release and downstream sEV processing.

### sEV origin affects uptake mechanism and in turn recipient cell response

**Figure 5:**
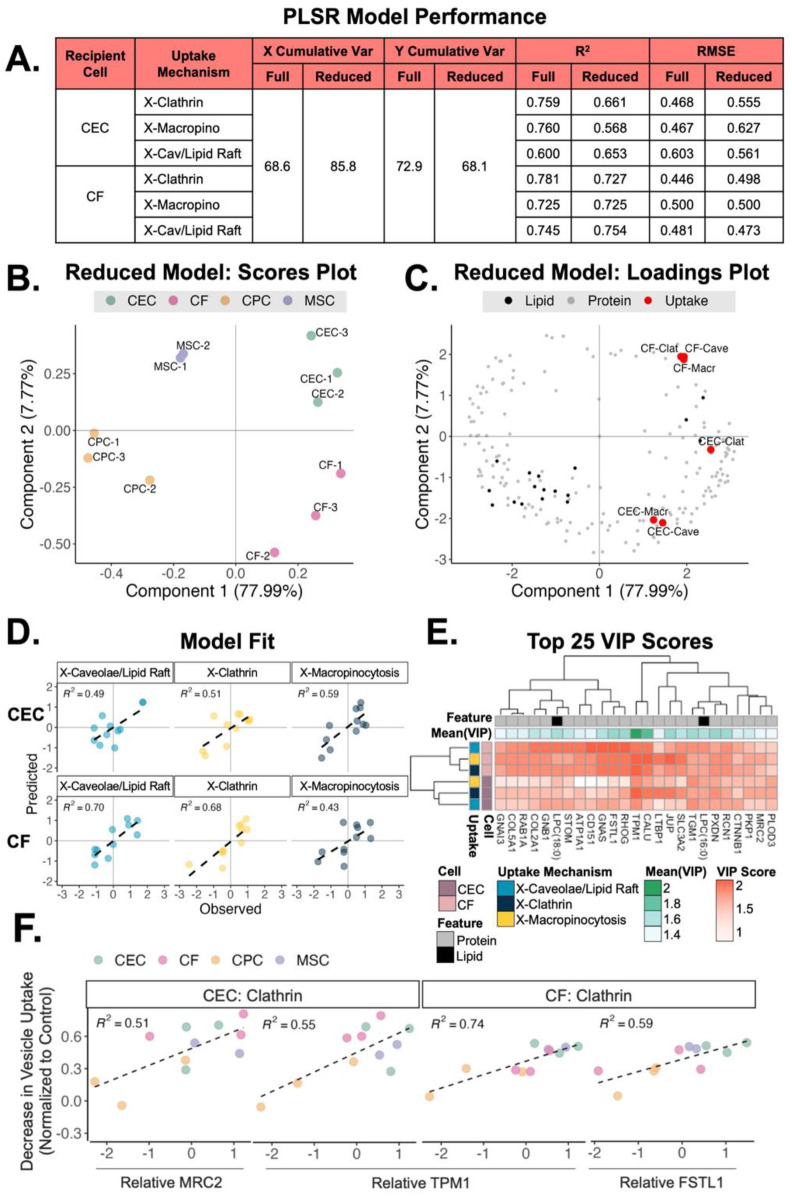
Relationship between sEV origin, uptake mechanism and recipient cell type. **(A)** Table of slope (R^2^) and error (RMSE) for a PLS regression analysis with full model (711 combined protein and lipid features) and a reduced model (top 303 protein and lipid VIPs). Only normoxic protein and lipid features used. **(B)** Scores plot of clustering from the 4 different sEV groups across component 1 (78% variance) and component 2 (8% variance). **(C)** Loadings plot of sEV proteins and lipids mapped with the CEC and CF uptake mechanisms. **(D)** Predictions for hypoxic conditioned sEV uptake (testing data) vs. observed values. **(E)** Heatmap of the top 25 VIP protein/lipid features (mean VIP score across all conditions) by uptake mechanism and recipient cell type. **(F)** Scatter plots of vesicle uptake by recipient cell type with relative MRC2, TPM1 and FSTL1 protein expression. Scatter plots for complete top 25 VIPs is supplementary document. MRC2 = mannose receptor C type 2; TPM1 = tropomyosin-1; FSTL1 = follistatin-like protein 1.

After exploring the variations in lipid and protein profiles from sEVs of different origins, we next sought to understand the relationship between the sEV membrane profiles and uptake mechanism. For this, a PLS regression model was developed from the normoxic samples’ protein and lipid profiles (X; 711 total features) and uptake mechanism data (Y; 6 outcomes). Using PLS, we reduced these large data matrices to 3 components, or dimensions, and performed regression to understand the link between ‘omics and uptake. The resulting full model described 69% of the variance in lipid/protein features (X) and 73% of the variance in sEV uptake (Y). The coefficient of determination (R^2^) for each of these uptake outcomes in this model was between 0.6 and 0.78 (Figure 5A).

To improve model performance and understand the most important variables contributing to the projection (VIP) we reduced the model to the top 303 VIP lipids/proteins (score > 1). The reduced model explained greater cumulative variance in lipid/protein features, as expected. Importantly, the reduced model improved R^2^ and RMSE metrics for X-Cav/Lipid Raft outcomes. The reduced model was subsequently used for further analyses.

Using this supervised approach, the scores plot from the reduced PLS regression model showed that the sEV samples cluster distinctly by parent cell type (Figure 5B). Further, the loadings plot shows that the uptake mechanisms cluster by recipient cell type across component 2, with delivery to CECs in quadrant 4 and delivery to CFs in quadrant 1 (Figure 5C). To understand performance and general applicability, we applied the reduced model (trained on normoxic samples) to a testing set of our hypoxic sEV data (Figure 5D). The model demonstrated good uptake predictions with R^2^ between 0.43 and 0.7. Finally, we investigated the top 25 VIP proteins and lipids in the reduced model (Figure 5E). The difference in VIP score of each feature in each uptake mechanism highlights the importance of LTBP1 (latent transforming growth factor beta binding protein), SLC3A2, and CALU (clusterin). We further explored the directionality of these top 25 VIPs to see which features were associated with uptake when the 3 uptake pathways were inhibited (Figure 5F and Supplementary Figures S6-S30). Evidently, with increasing levels of MRC2 and TPM1, there is a larger decrease in vesicle uptake by CECs when administering clathrin inhibitor. A similar pattern is noted for CFs with increasing levels of TPM1 and FSTL1. To validate these findings, the uptake of MSC/CPC sEVs by CF/CEC with either MRC2 or FSTL1 blocked was assessed (Supplementary Figure S31). Reduced uptake of MSC and CPC sEVs was observed when either protein was blocked. Further, to elucidate the functional impact of these proteins, a tube formation assay was performed with MSC/CPC sEVs on CECs with MRC2 inhibition (Supplementary Figure S32). As suggested by the model, the inhibition of MRC2 significantly reduced tube formation parameters compared to no inhibition. Together, these data validate the findings of the model and suggest that membrane composition may aid with preferred uptake method and downstream effect, especially for clathrin-mediated vesicle uptake.

## Discussion

sEVs membranes consist of a complex of lipids and proteins, with composition dependent on the vesicle's parent cell type. As sEVs are important mediators of cell-cell communication, and uptake of sEVs depends on interaction of the sEV membrane with the recipient cell, there is value to understanding the variations in sEV membranes and their effect on uptake. In this work, we explore the diverse lipid and protein profiles of sEVs derived from four cardiac-relevant cell types: CPCs, MSCs, CECs and CFs. We establish that sEV parent cell type is one of the primary sources of variance in membrane composition, and that clathrin-mediated uptake varies the most across parent cell types with macropinocytosis varying when sEVs are treated to CEC recipient cells. We then connect the lipid-protein profiles to uptake mechanism and recipient cell type, finding that for a given recipient cell type, the membrane profile for each uptake mechanism is distinct. Together, these data underscore the importance of understanding the membrane profile and its importance in sEV function.

For this study we selected four cardiac-relevant cell types, namely CPC, MSCs, CECs and CFs. This choice was made to include 2 key stem/progenitor cell types as well as an endothelial and fibroblast cell type w35hich are critical during cardiac repair and remodeling. Given the extensive technical challenges with isolating sufficient sEVs from non-proliferating cultured cardiomyocytes (CMs) for proteomic and lipidomic analysis, CMs were excluded from this study. Despite this, stem/progenitor cells and endothelial/fibroblast cells have well-established roles in cardiac repair, thereby garnering significant interest as sources for sEV therapies. Further, we specifically chose rat CECs and CFs for this study. In the future, this allows us to conduct downstream *in vivo* studies in rat cardiac-models by leveraging relationship of the membrane proteins/lipids to sEV uptake that was unveiled in this study. Further, this will allow targeted engineering of sEV membrane for cell-specific sEV uptake *in-vivo* to expand upon this work.

One source of variation in sEV function is oxygen conditioning. Prior work from our lab and others have shown that hypoxic conditioning of CPCs and MSCs can generate more reparative sEVs in the context of cardiac repair (25, 26, 33) Although the role of the cargo (specifically microRNA) in the pro-reparative effects has been well explored, the potential role of the membrane has not been examined. Here, we isolated sEVs from both CPCs and MSCs in normoxic and hypoxic conditions to assess variation in the membrane profiles and whether this variation could affect uptake and in-turn function. No significant differences were observed in the lipid or protein profiles of the sEVs or in the uptake mechanisms utilized by the normoxic and hypoxic sEV counterparts. This reiterates that the cargo may be the primary mediator of the observed differences and not the membrane or uptake efficiency. However, patient-derived factors may drive differences in sEV efficacy, shown from previous studies. In the case of CPC sEVs, responses were conditional upon age, with hypoxic conditioning affecting sEV potency in CPCs derived from older patients (32). In addition, the duration of hypoxic incubation could affect changes in protein/lipid composition with longer incubation periods having a significant effect. Therefore, there is value in exploring both hypoxia incubation duration and age-dependency on the sEV membrane profile in the future.

To understand the role of different uptake mechanisms we used small-molecule inhibitors of each pathway. Upon quantification of the uptake efficiency, in some cases, inhibition of the macropinocytosis pathway seemed to further increase sEV uptake beyond the extent of sEV-only uptake (without any inhibitors). Although this appears counter-intuitive, it should be noted that the sEV-only uptake does not represent uptake of 100% of loaded sEVs but rather the *maximum* uptake without any inhibitors. Therefore, the greater-than-one uptake when macropinocytosis is inhibited is still realistic. Despite this, the improvements in CPC- and MSC-sEV uptake by CECs and CFs, respectively, upon inhibition of macropinocytosis is interesting. Prior studies assessing uptake in cancer cells found that inhibition of macropinocytosis reduced sEV uptake (11, 34). However, it's established that the capacity of clathrin-independent uptake methods, which includes macropinocytosis, can largely vary upon differences in experimental procedures (e.g., serum starvation), cell types, and cell physiological states (e.g., cell confluency) (35). Further, we chose chemical small-molecule inhibitors for this study and validated their efficiency at inhibiting albumin and transferrin uptake. However, it should be noted that chemical inhibitors often have broad-targets and could partially affect other mechanisms of action as well. In addition, though several washes were performed to remove surface-bound sEVs, part of the observed enhanced “internalization” with micropinocytosis inhibition may be an artifact of sEV accumulation on the cell surface. Taken together, these findings suggests that the increase in uptake of certain sEVs with macropinocytosis inhibition is realistic and may be inducing a compound effect that can be further explored.

Beyond understanding the role of sEV origin on uptake mechanism, we also explored the lipid and protein profiles of the different sEVs and their role in uptake by CECs and CFs. Although few, other studies investigating sEV membrane lipids collected conditioned media over several days, whereas in our case, with the hypoxic conditioning, we isolated conditioned media after 12 h (36, 37). This reduced the concentration of sEVs in our studies. Consequently, despite successfully obtaining lipid features, after downstream processing, the unique lipid features were too few to establish robust pathway analysis. This did not affect the scope of our study as we focused on the combinatorial effects of the membrane lipids and proteins, but as sEV isolation and concentration techniques improve, the scope to explore membrane lipids pathways like we did with proteins could provide more unique insights.

Finally, we established that clathrin-mediated uptake has more distinctly upregulated proteins (MRC2, TPM1, FSTL1) compared to caveolae- or macropinocytosis-mediated uptake. Further, some of these proteins vary by CEC or CF recipient cell. MRC2 is important in CF and CECs for TGF-b activity (38). A possible therapeutic target during cardiac hypertrophy, it is involved in cardiac extracellular matrix remodeling through collagen degradation. Interestingly, MRC2 receptor usually leverages clathrin-coated pits for the cargo transport (39), so understanding its role in vesicle uptake could improve targeted vesicle delivery. TPM1 is a well-established component of the sarcomere which aids actin-myosin interaction. It plays an important role in cardiogenesis and congenital defects (40). FSTL1, which we observed more highly expressed in CFs-Clathrin pathways, is expressed after myocardial infarction by CFs and myofibroblasts and is crucial during initial cardiac repair (41). Given the nascent field of understanding sEV composition in uptake, there isn't a well-explored connection between these proteins and vesicle uptake, but some of the proteins are highly associated with cardiac function and cardiac pathophysiology. Therefore, further investigation into the top VIPs highlighted in this study can help develop tailored sEVs for targeting specific CVDs.

## Conclusion

In this study we investigated the role of sEV protein/lipid composition on the uptake mechanism of sEVs (from four cell types) by recipient cells. This work has furthered our understanding of cardiac-relevant sEV composition and showcased that variations in the sEV protein/lipid content could directly impact the sEV uptake pathway. It has helped to foray into vesicle components and their effect on uptake, specifically within the cardiovascular space. This field of work helps us understand inherent sEV patterns with uptake and can aid with tailoring vesicle-therapies for selective and targeted delivery in the future.

## Data Availability

The raw data supporting the conclusions of this article will be made available by the authors, without undue reservation.
