# Supplementary material for: Composition of cardiac-derived extracellular vesicles changes with vesicle origin and determines uptake
Source: Front Cardiovasc Med. 2025 Sep 15;12:1565104. doi: 10.3389/fcvm.2025.1565104 (PMC12477219; doi:10.3389/fcvm.2025.1565104)
Supplement: Supplementary file 1 [file Datasheet1.pdf]

Supplementary Figure 1: Immunoblot of sEV markers

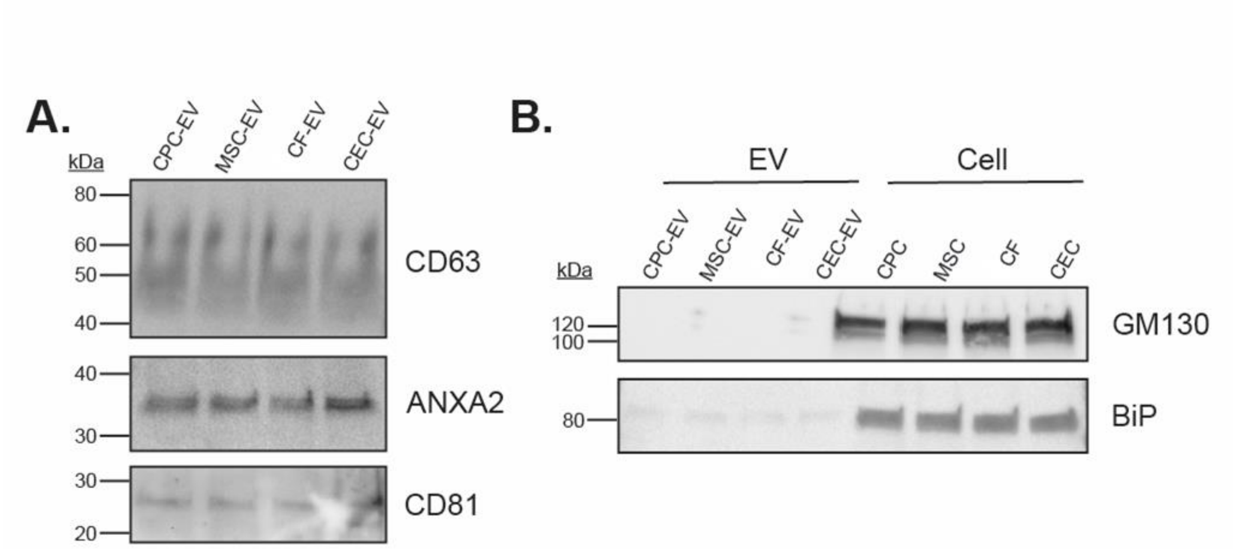

Supplementary Figure 1: Immunoblot for all four sEV groups and corresponding parent cell lysates for (A) positive markers for sEVs, CD63 (+), ANXA2 (+) and CD81 (+) and (B) negative markers for sEVs, GM130 (-) and BiP (-).

Supplementary Figure 2: sEV size and concentration by oxygen condition

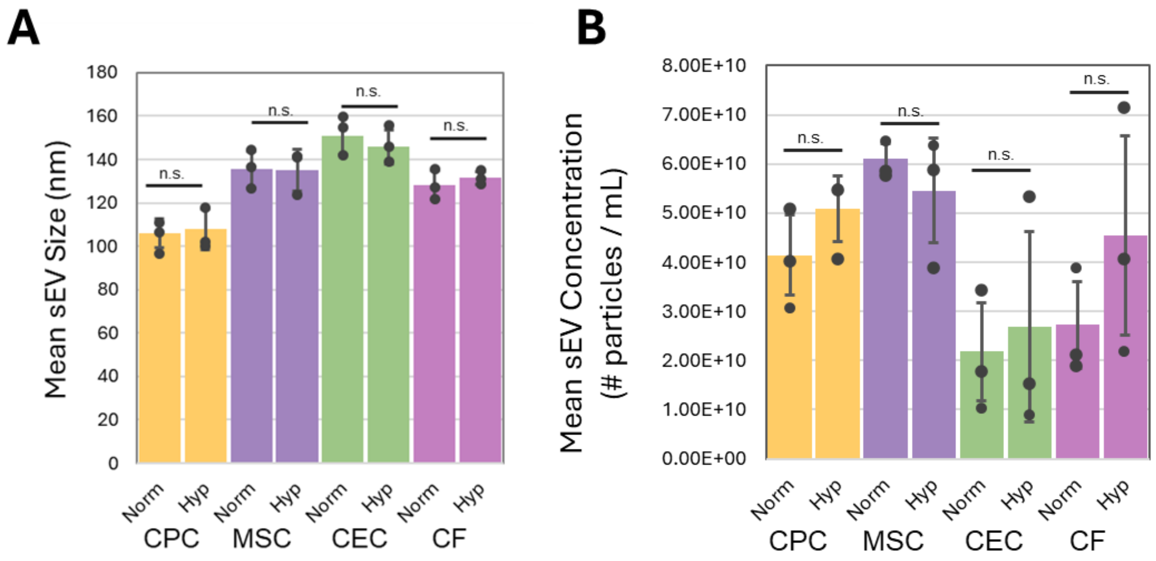

Supplementary Figure 2: sEV mean size and concentration by oxygen condition. (A) Mean size of sEVs and (B) Mean concentration of sEVs in normoxic and hypoxic conditions. Data shows no significant difference in sEV size or concentration based on oxygen condition within each cell type. Mean ±SEM. Significance within each parent cell type (e.g. CPC) was tested with a two-sided T-test. n.s. = no significance. Norm=normoxia, Hyp=hypoxia.

<u>Supplementary Figure 3: Flow Cytometry gating strategy</u>

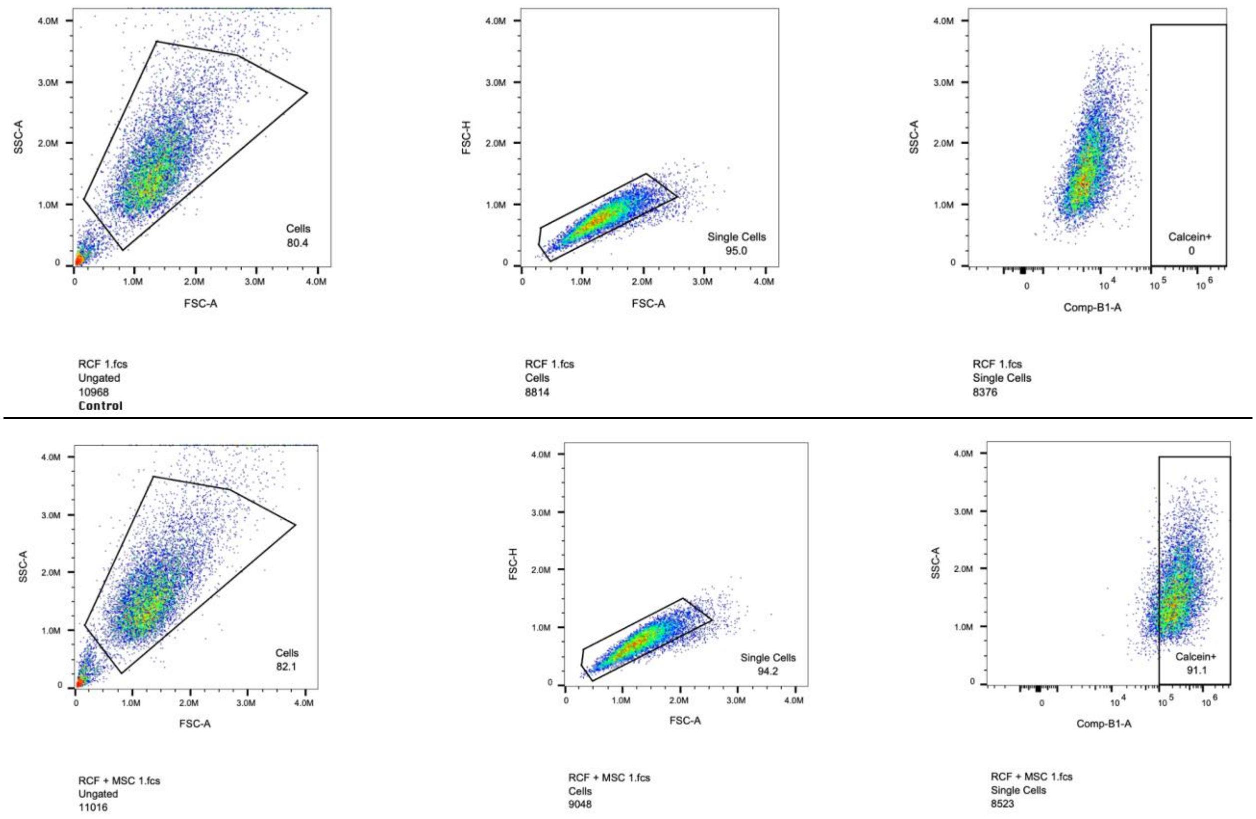

Supplementary Figure 3: Gating strategy for flow cytometry cell quantification. FSC-A vs SSC-A (exclude debris), FSC-A vs FSC-H (exclude aggregates, doublets etc), Comp-B1-A vs SSC-A (exclude calcein-negative cells). Top row: Control cell only, used to determine Comp-B1-A gating location; Bottom row: cells + MSC sEVs.

Supplementary Figure 4:

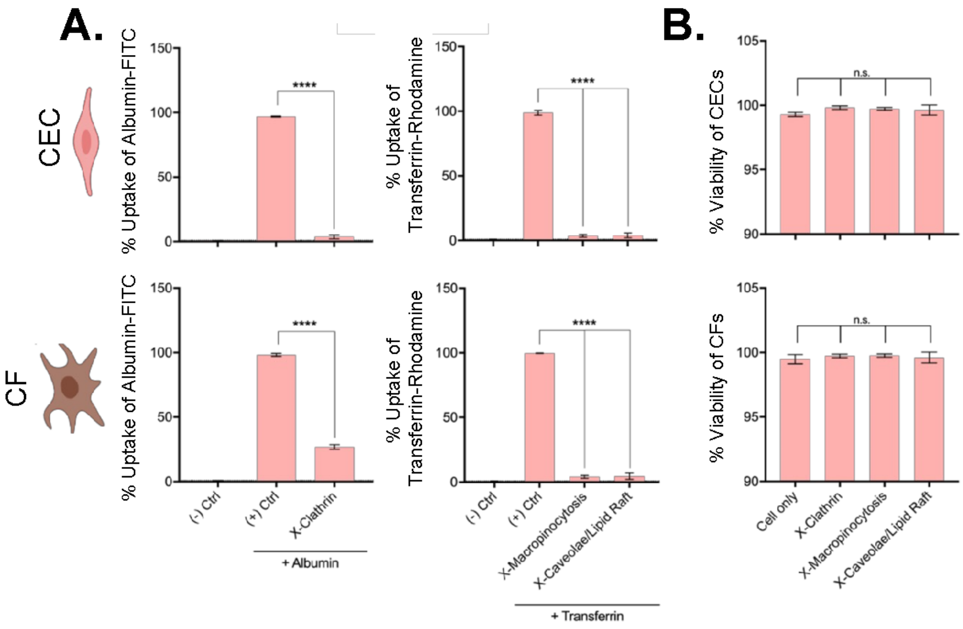

Supplementary Figure 4. Small molecule inhibitor potency assessment for sEV uptake pathway inhibition. (A) Potency of the selected concentration of each small molecule inhibitor (ref. Table 1) to inhibit the corresponding uptake pathway against albumin and transferrin. Pitstop-2 (clathrin inhibitor), Nystatin (caveolae/lipid raft mediated uptake inhibitor) or Amiloride (Na+/H+ pump mediated macropinocytosis inhibitor). Albumin uptake is clathrin dependent wherease Transferrin uptake is clathrin-independent therefore both used to confirm appropriate inhibition by each inhibitor (B) Effect of small molecular inhibitor on CEC and CF cell viability. Mean±SEM. Significance was tested with one-way ANOVA with Tukey's post-hoc. n.s. = not significant. ****P<0.0001.

Supplementary Figure 5:

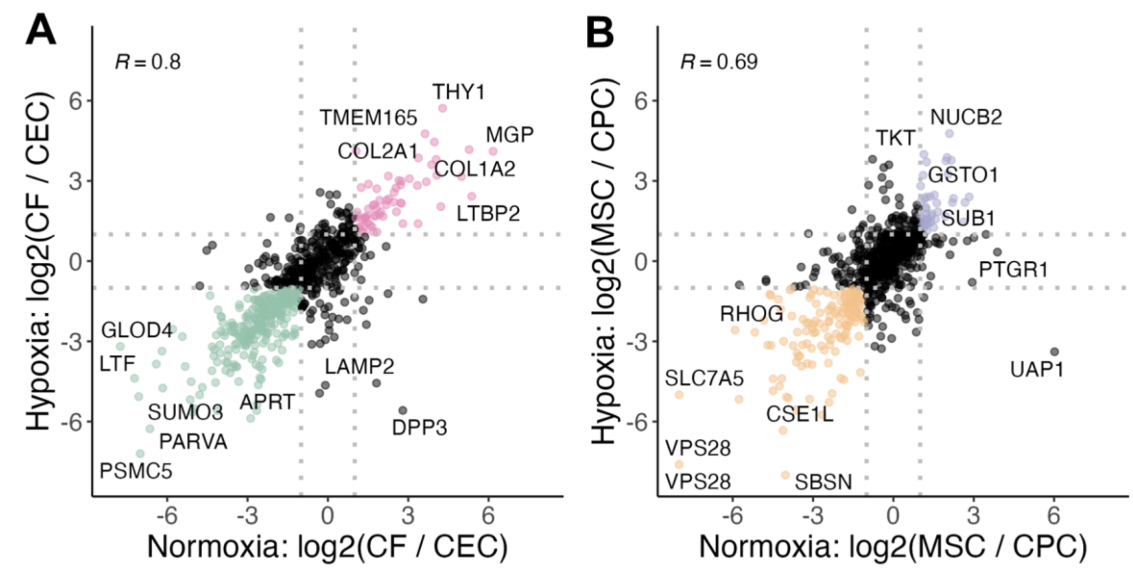

Supplementary Figure 5: Comparison of differentially abundant proteins between Normoxia and Hypoxia. Good agreement between oxygen conditions indicates differentially abundant proteins between the sEV types are similar when exposed to different oxygen conditions. (A) Log2FC of CF/CEC in Normoxic vs Hypoxic conditions. (B) Log2FC of MSC/CPC in Normoxic vs Hypoxic conditions.

<u>Supplementary Figures 6-30: Scatter plots for complete top 25 VIPs</u>
The following figures 6-30 represent the relative expression of the top 25 proteins across CEC/CF for each uptake inhibition pathway. These data represent the impact of each protein on each sEV uptake inhibition pathway. This allows an understanding of the specific relationship between the top VIPs and the corresponding uptake pathways and CEC/CF cell types. Some of these key relative protein expressions have been included in Figure 5, the rest are included in the figures below.

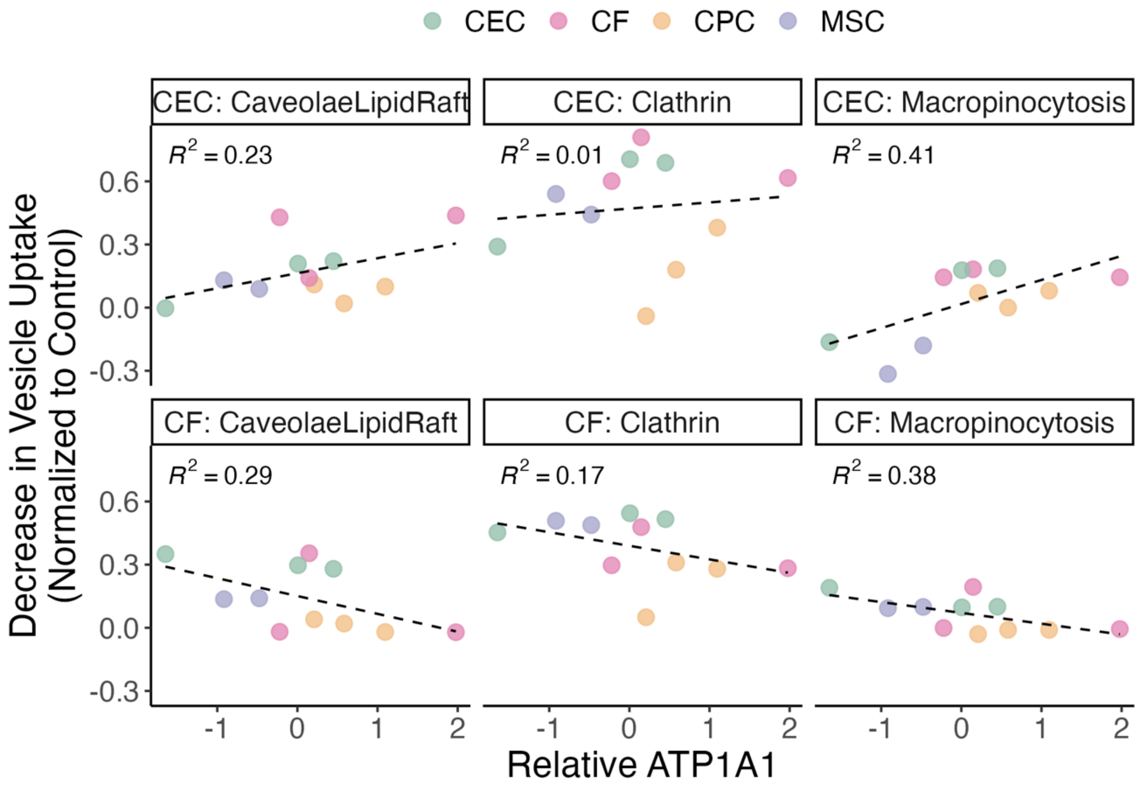

Supplementary Figure 6: Scatter plots of vesicle uptake by recipient cell type and uptake mechanism with increase in relative ATP1A1.

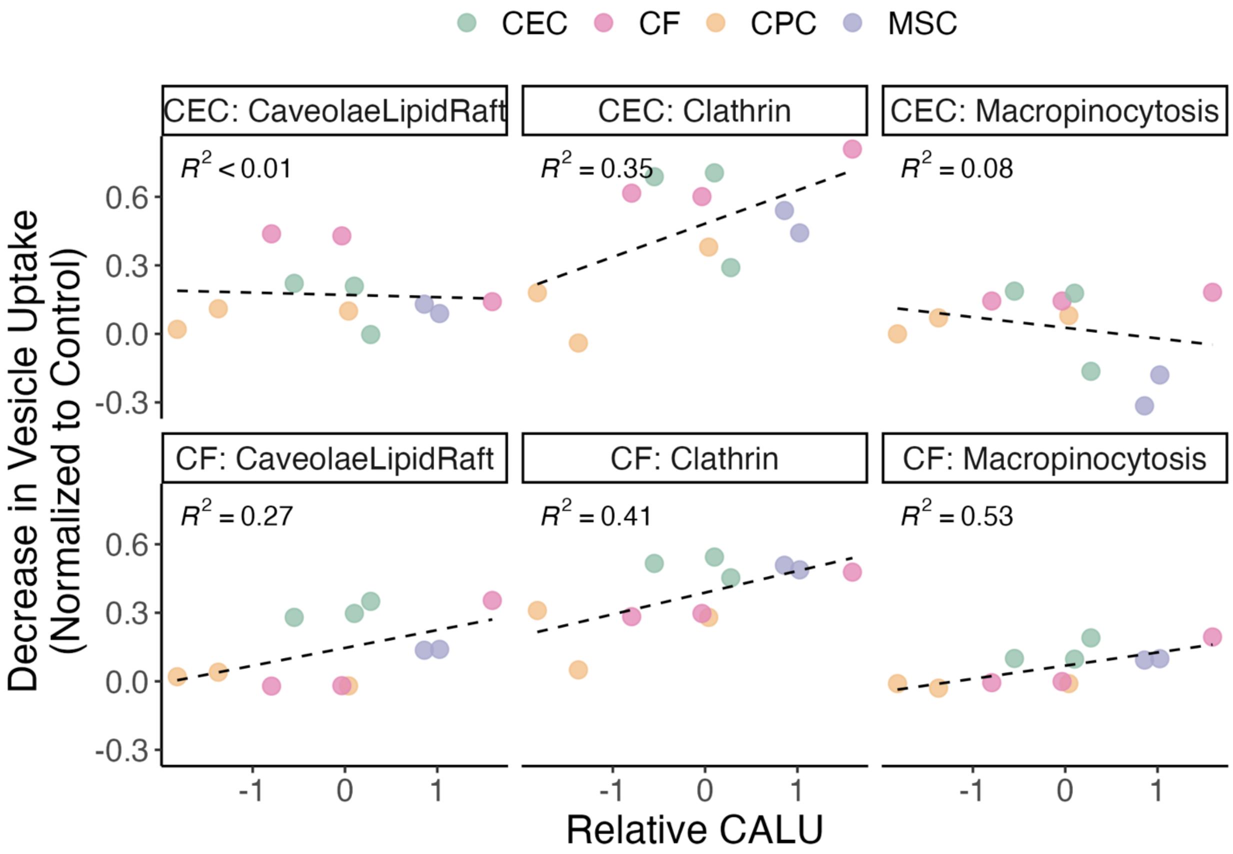

Supplementary Figure 7: Scatter plots of vesicle uptake by recipient cell type and uptake mechanism with increase in relative CALU.

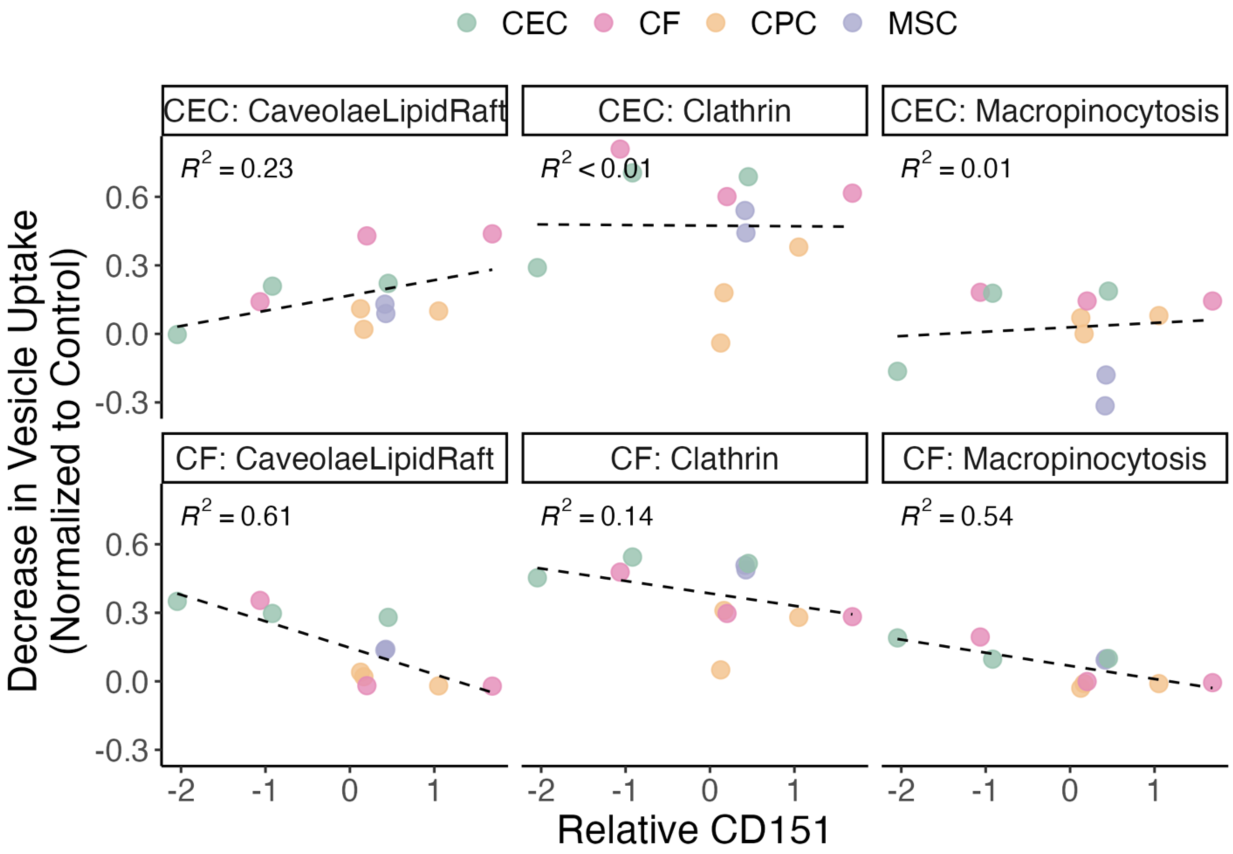

Supplementary Figure 8: Scatter plots of vesicle uptake by recipient cell type and uptake mechanism with increase in relative CD151.

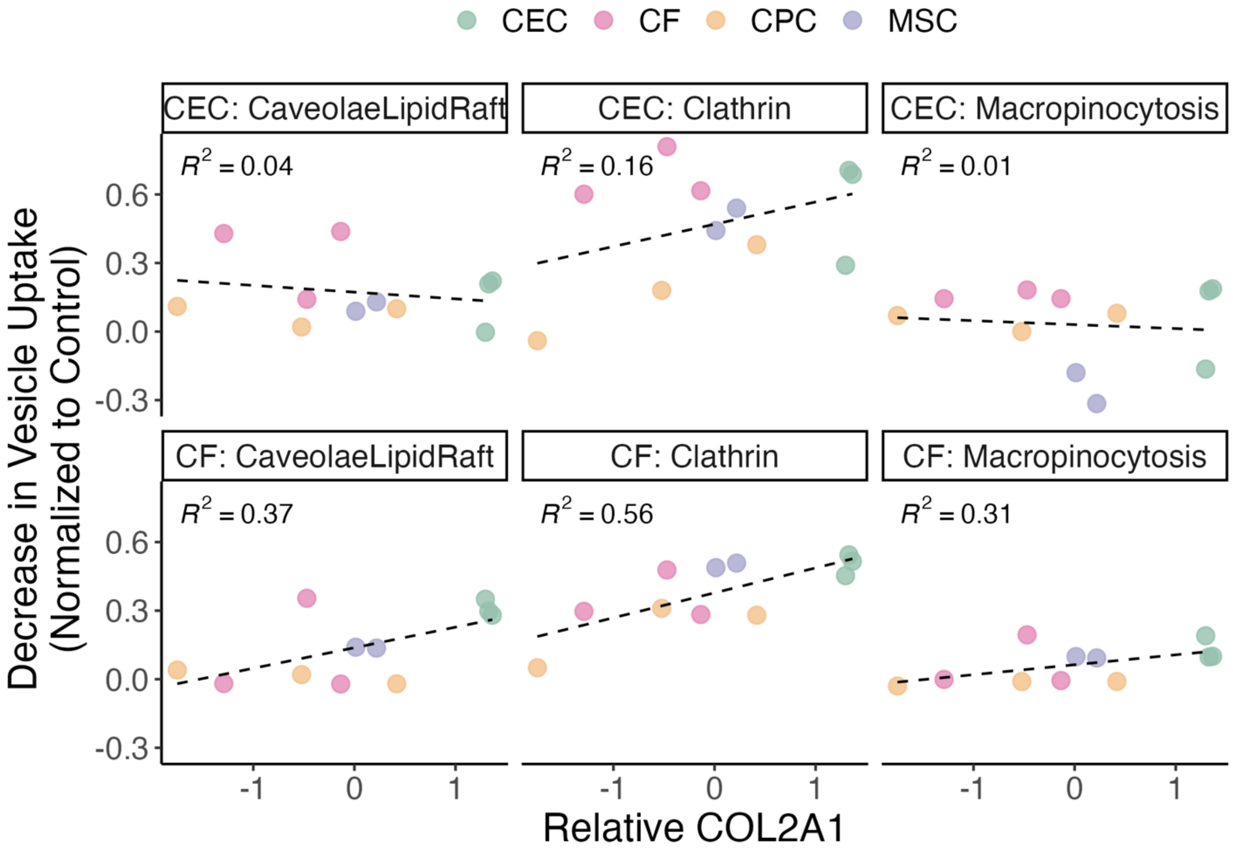

Supplementary Figure 9: Scatter plots of vesicle uptake by recipient cell type and uptake mechanism with increase in relative COL2A1.

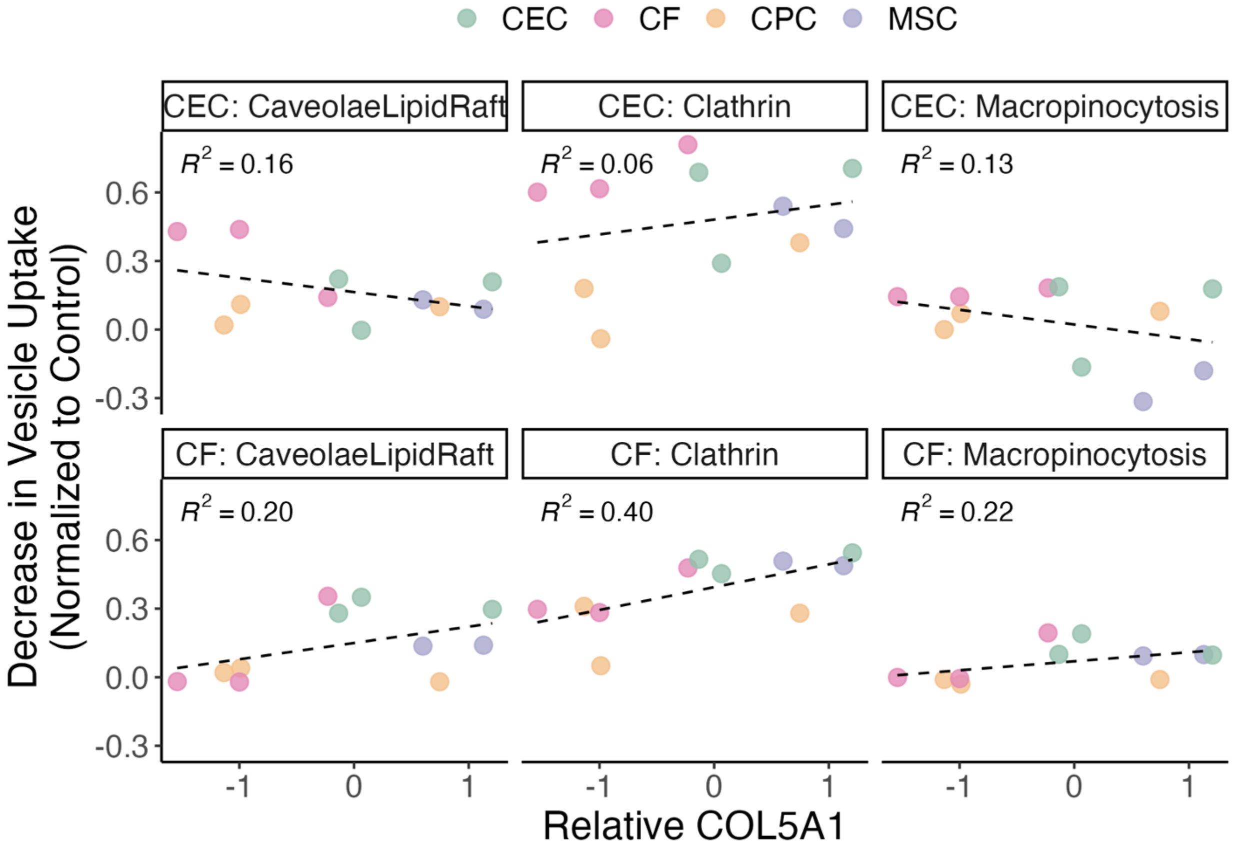

Supplementary Figure 10: Scatter plots of vesicle uptake by recipient cell type and uptake mechanism with increase in relative COL5A1.

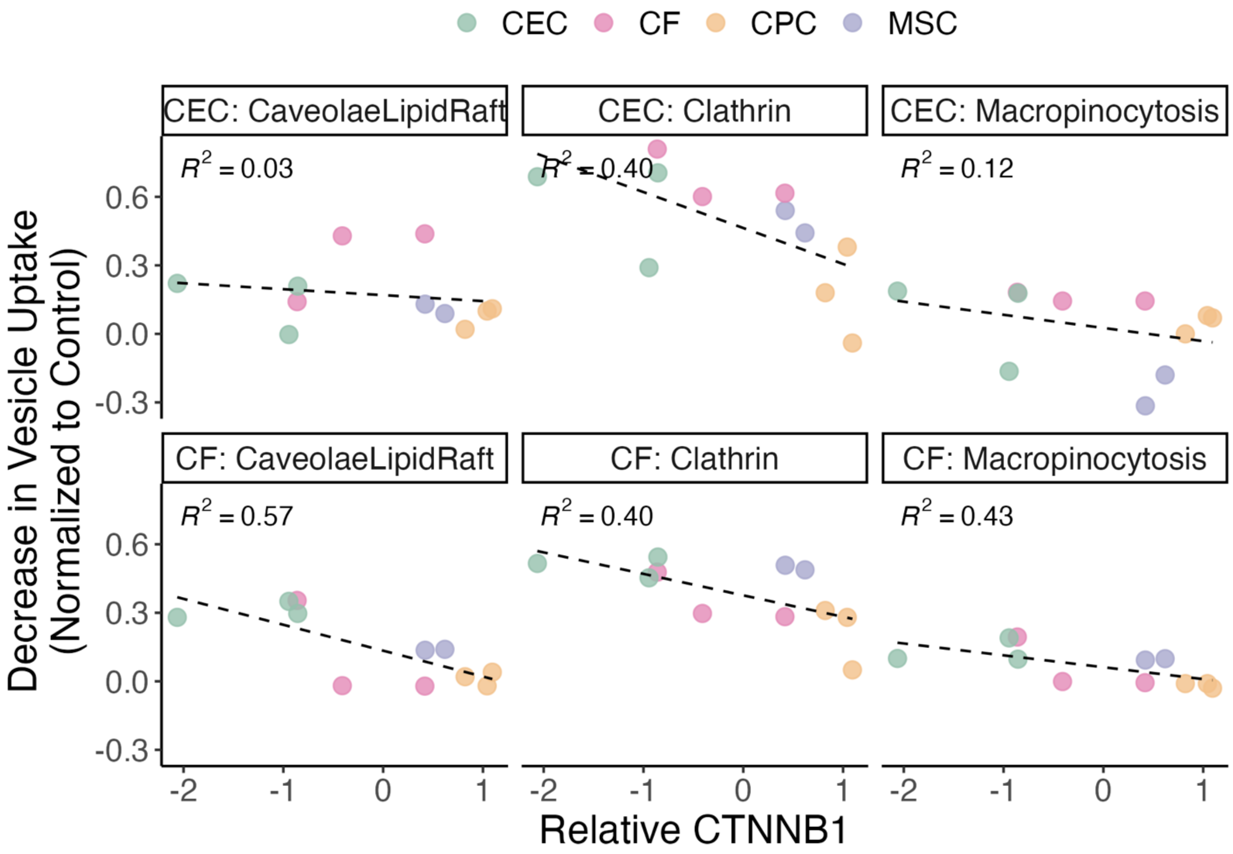

Supplementary Figure 11: Scatter plots of vesicle uptake by recipient cell type and uptake mechanism with increase in relative CTNNB1.

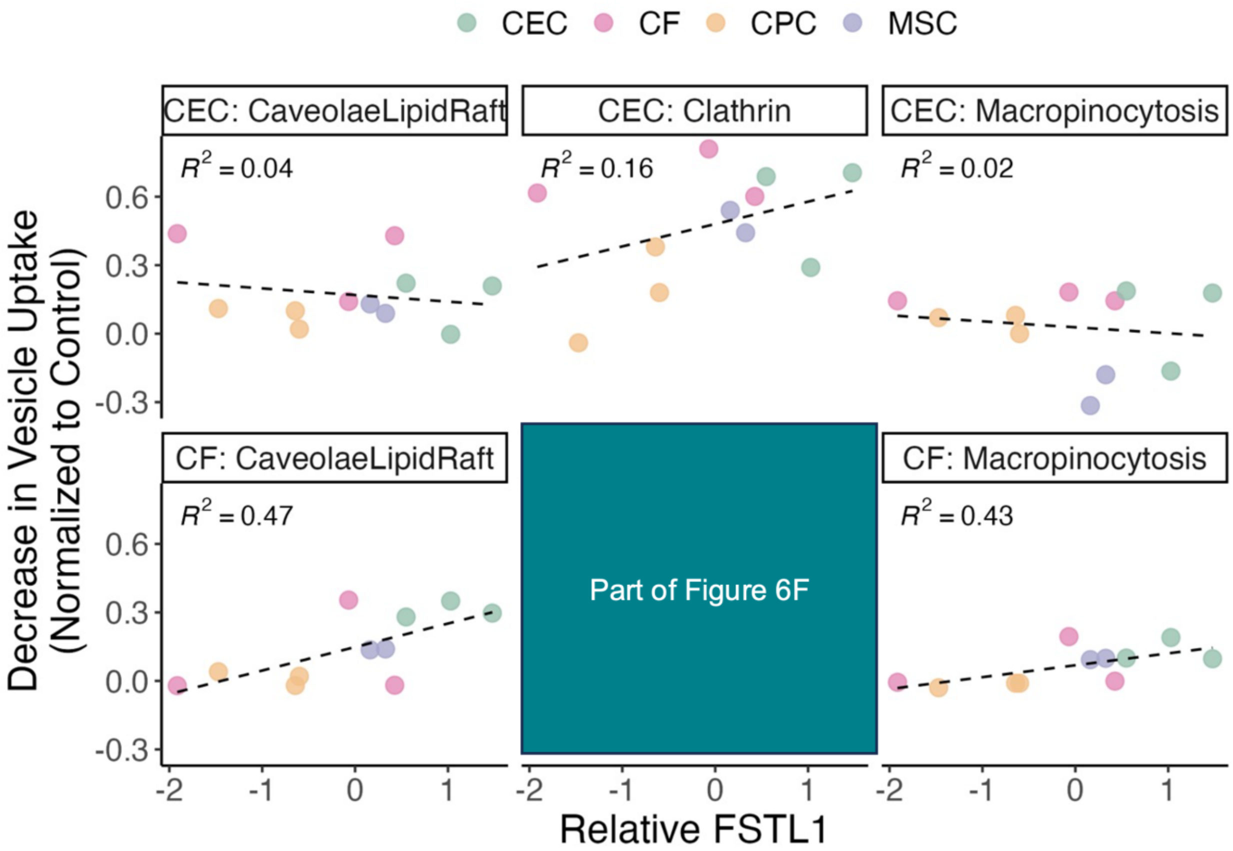

Supplementary Figure 12: Scatter plots of vesicle uptake by recipient cell type and uptake mechanism with increase in relative FSTL1.

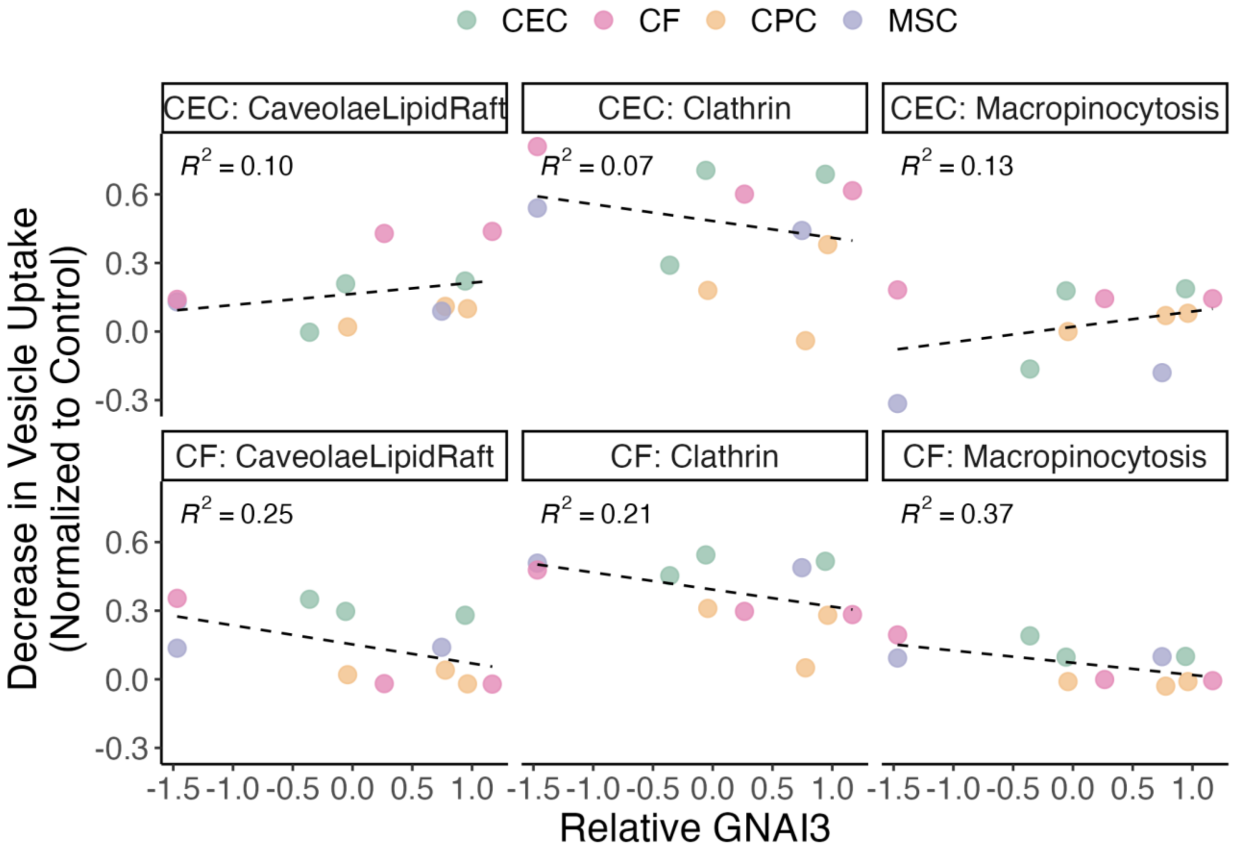

Supplementary Figure 13: Scatter plots of vesicle uptake by recipient cell type and uptake mechanism with increase in relative GNAI3.

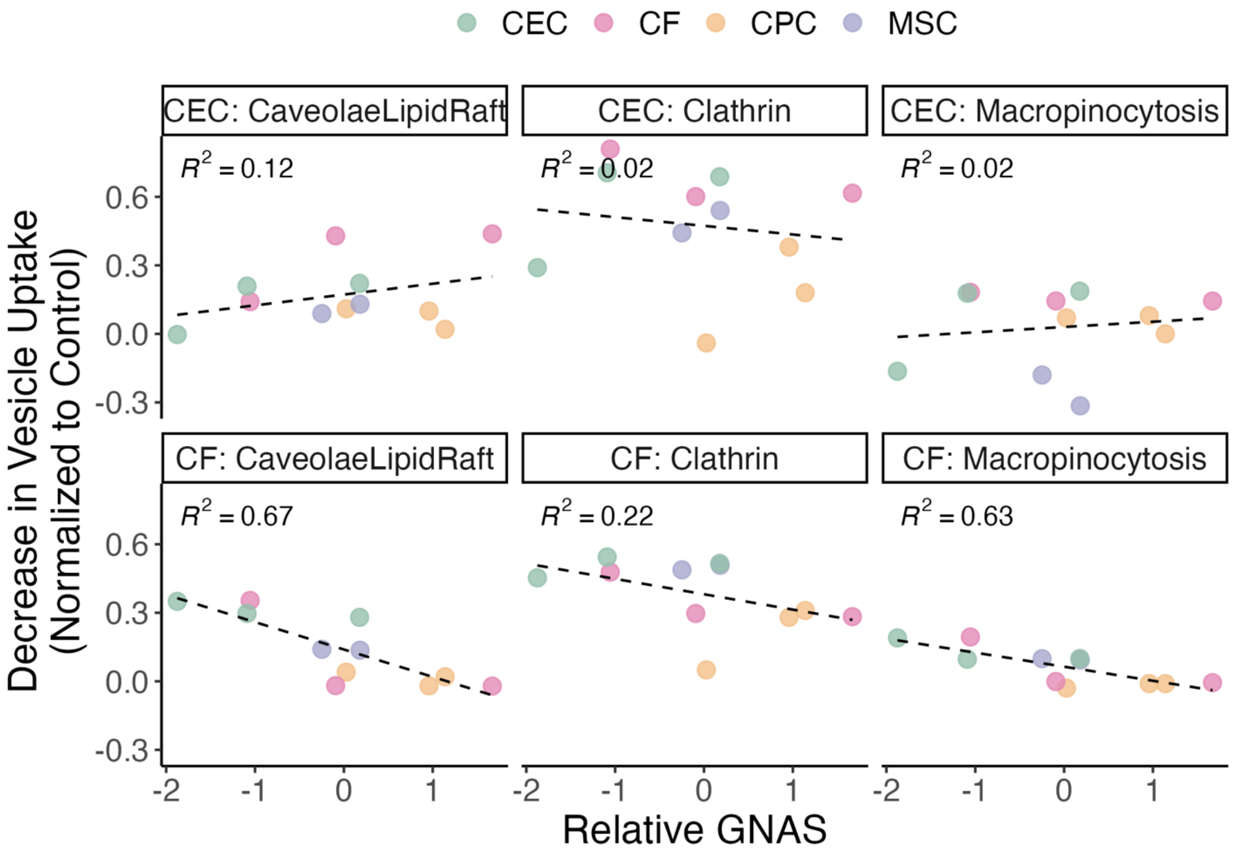

Supplementary Figure 14: Scatter plots of vesicle uptake by recipient cell type and uptake mechanism with increase in relative GNAS.

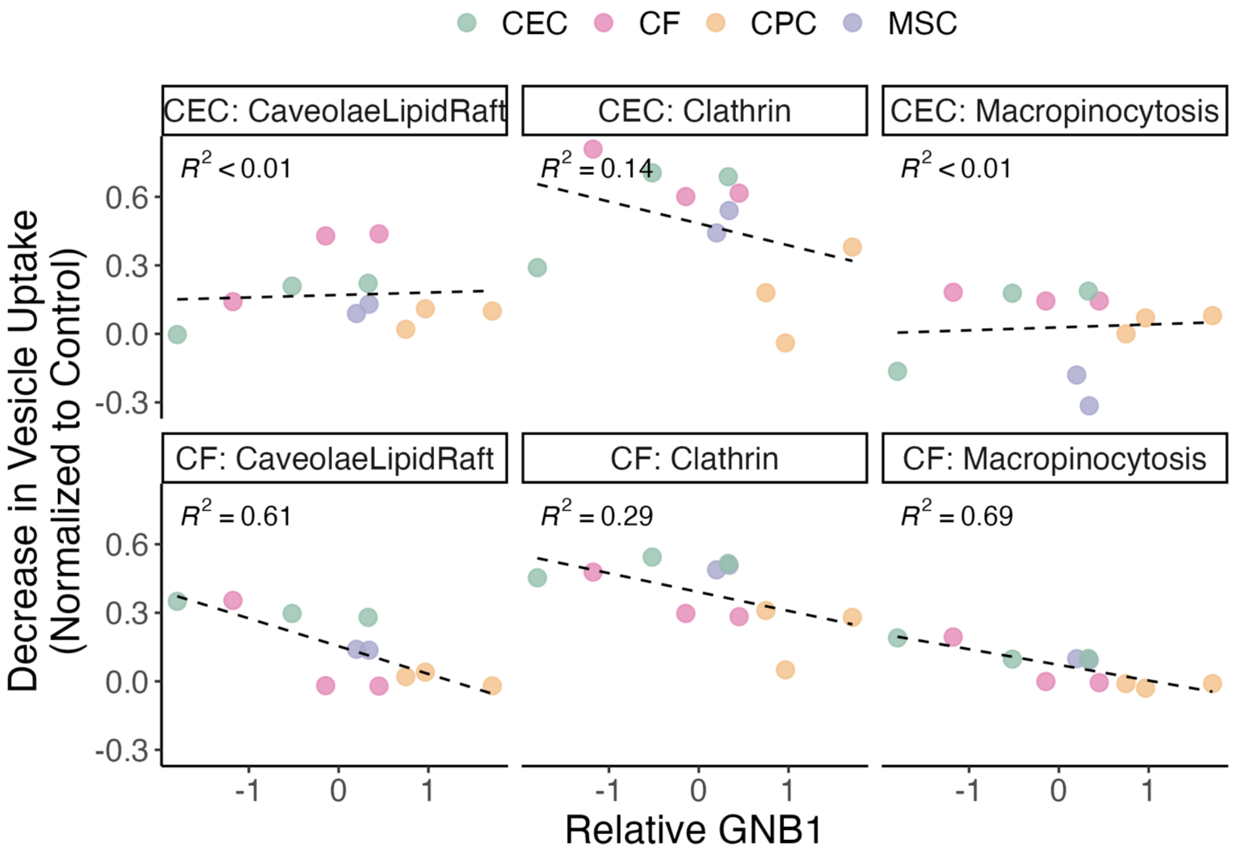

Supplementary Figure 15: Scatter plots of vesicle uptake by recipient cell type and uptake mechanism with increase in relative GNB1.

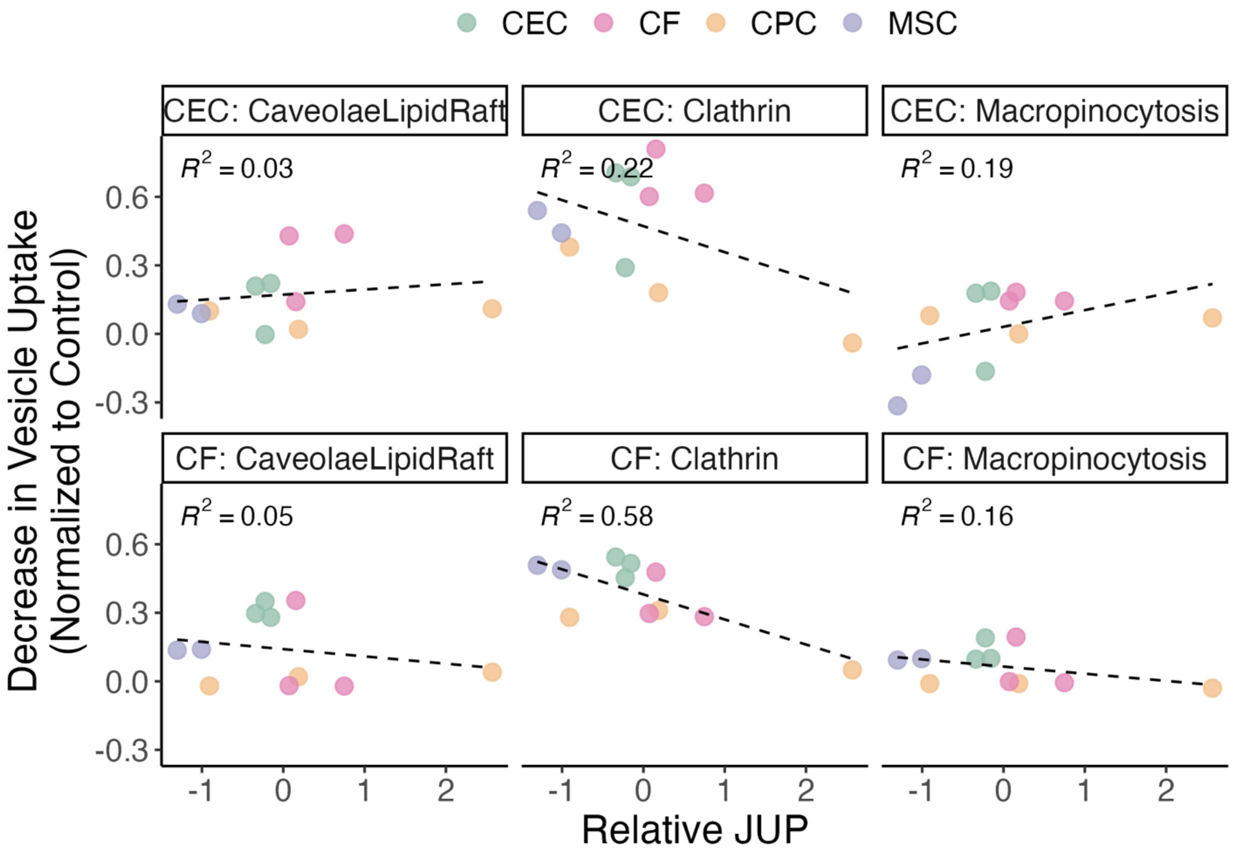

Supplementary Figure 16: Scatter plots of vesicle uptake by recipient cell type and uptake mechanism with increase in relative JUP.

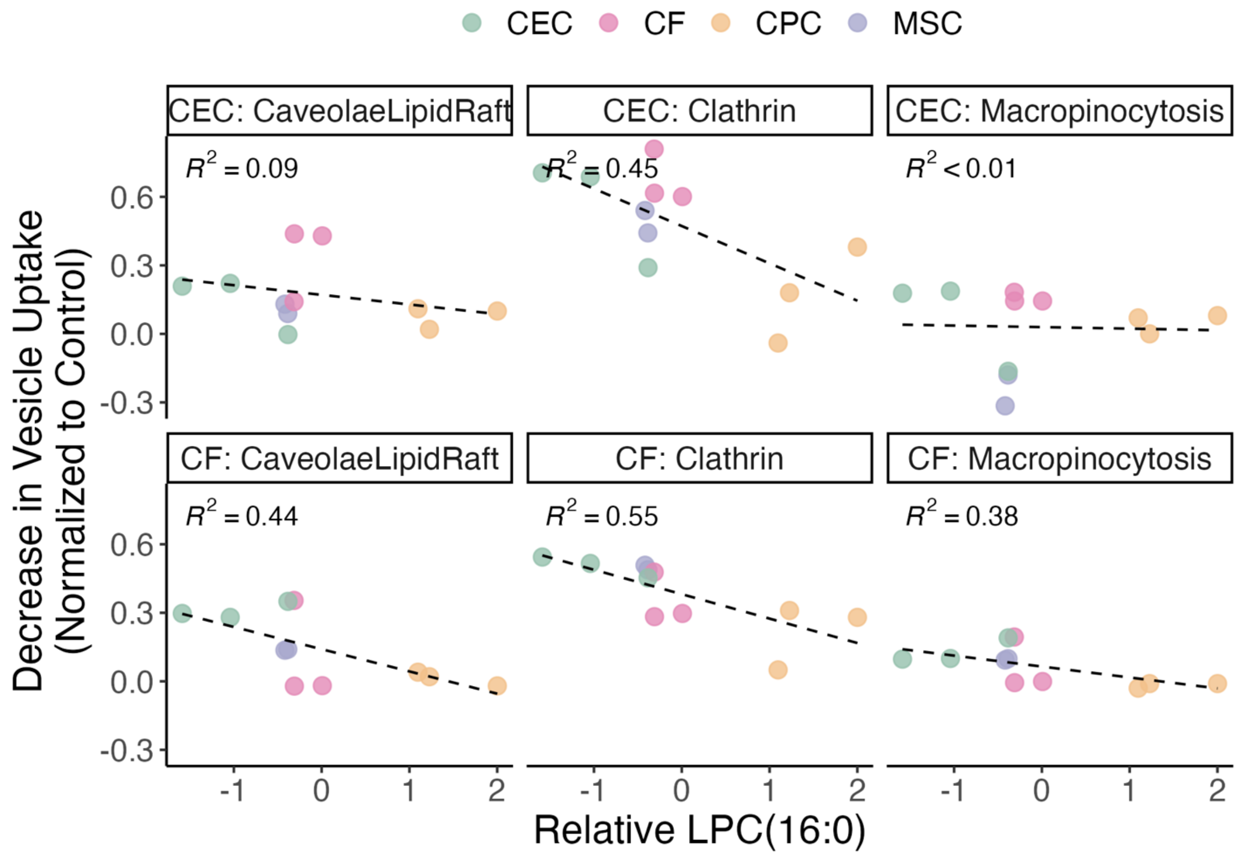

Supplementary Figure 17: Scatter plots of vesicle uptake by recipient cell type and uptake mechanism with increase in relative LPC(16:0).

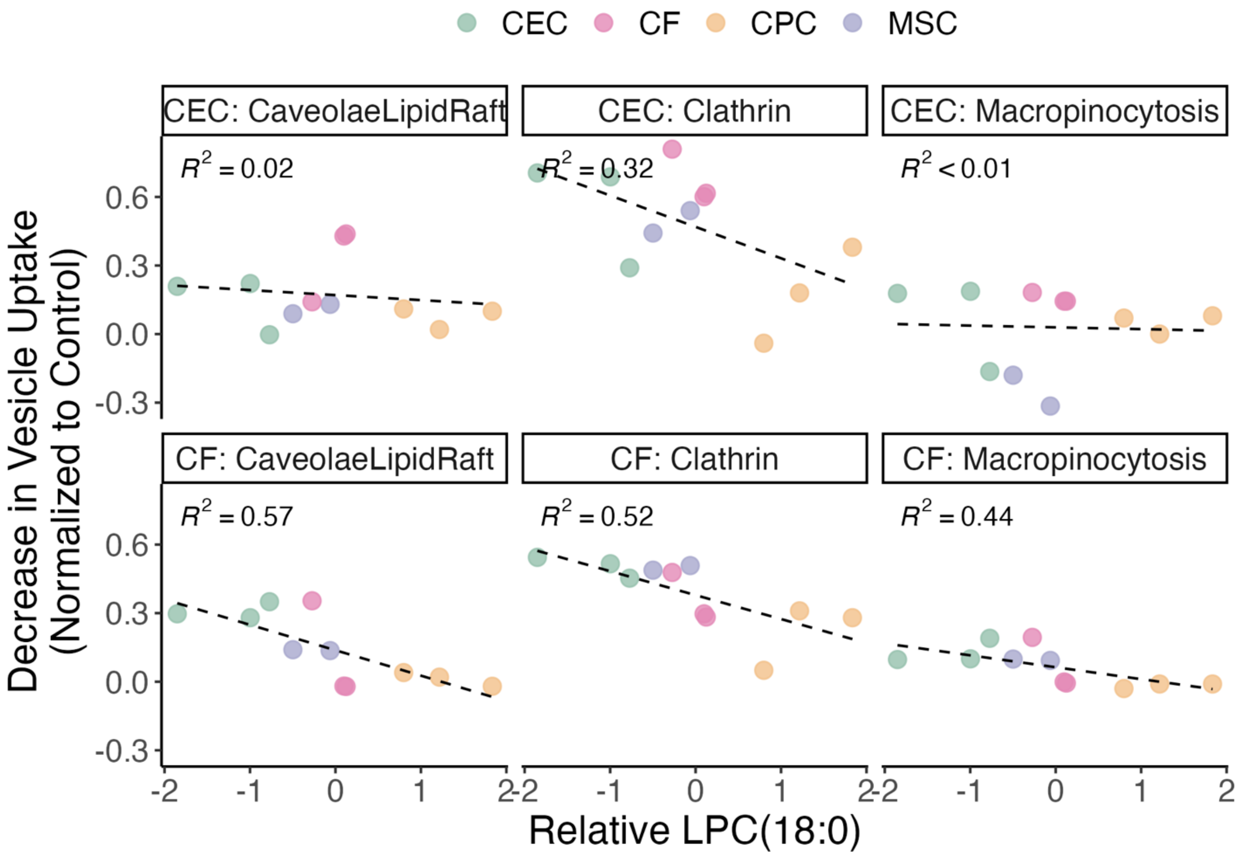

Supplementary Figure 18: Scatter plots of vesicle uptake by recipient cell type and uptake mechanism with increase in relative LPC(18:0).

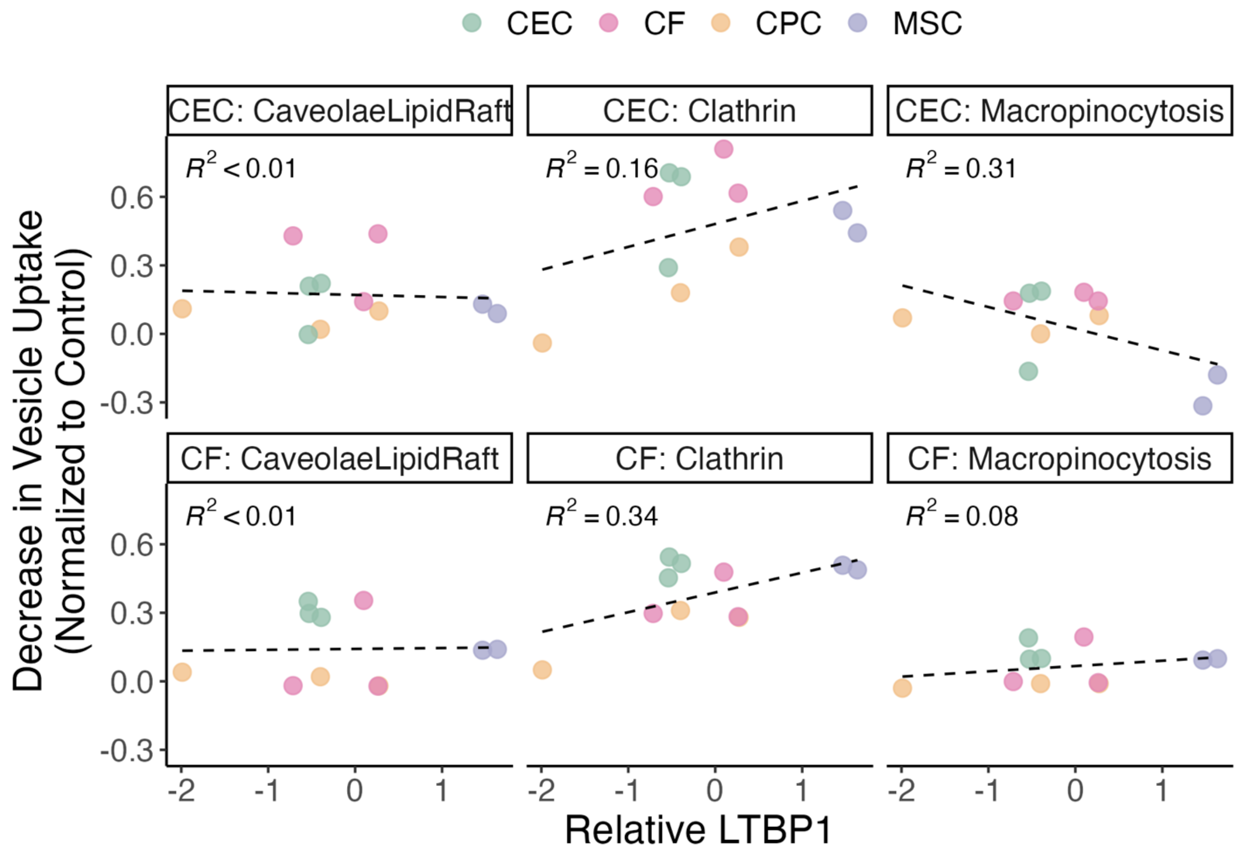

Supplementary Figure 19: Scatter plots of vesicle uptake by recipient cell type and uptake mechanism with increase in relative LTBP1.

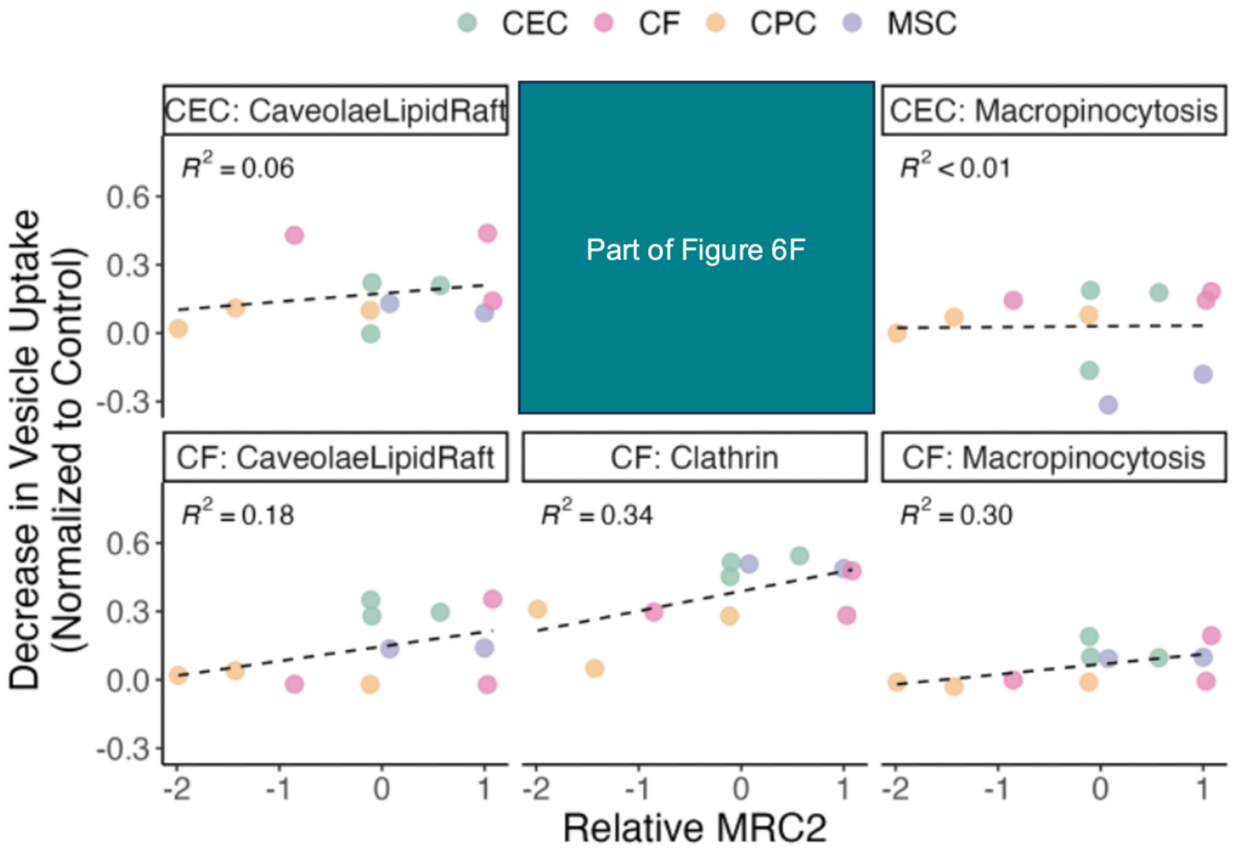

Supplementary Figure 20: Scatter plots of vesicle uptake by recipient cell type and uptake mechanism with increase in relative MRC2.

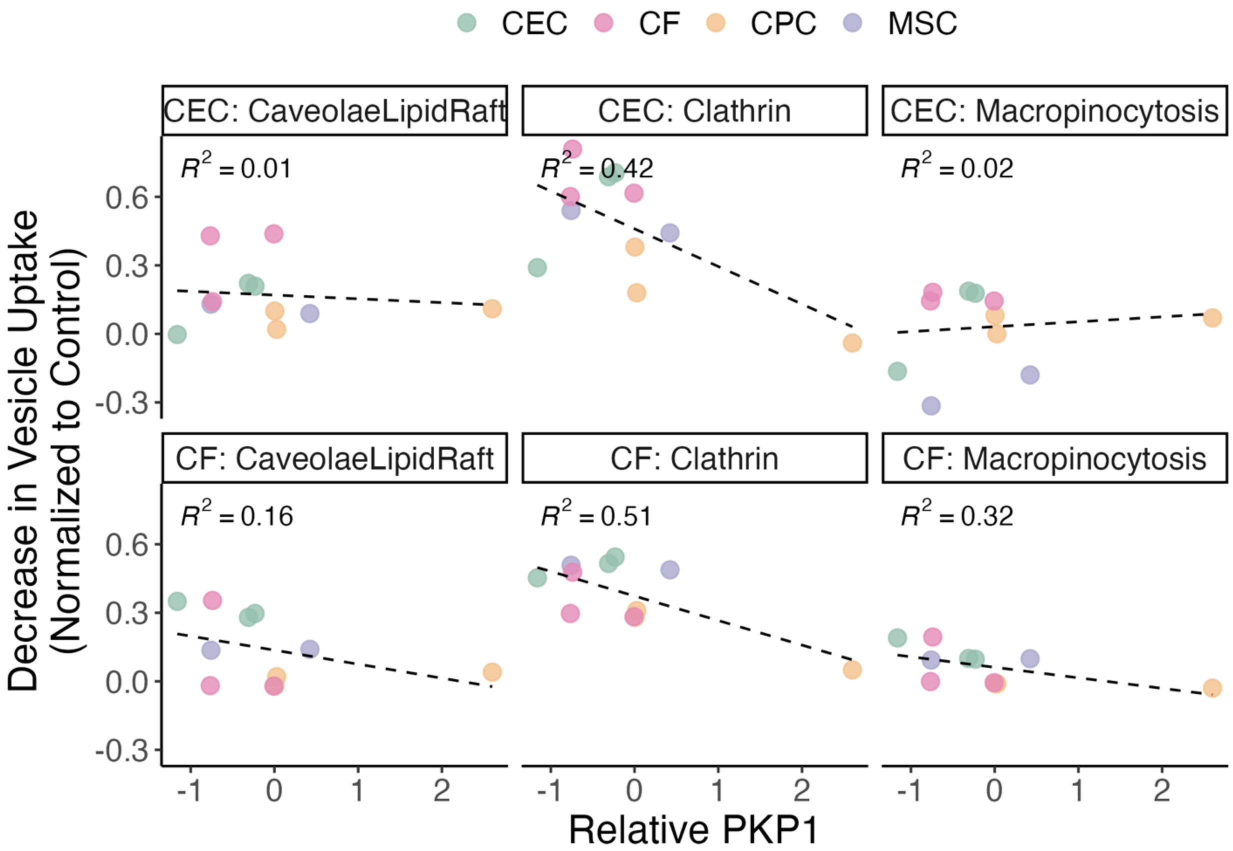

Supplementary Figure 21: Scatter plots of vesicle uptake by recipient cell type and uptake mechanism with increase in relative PKP1.

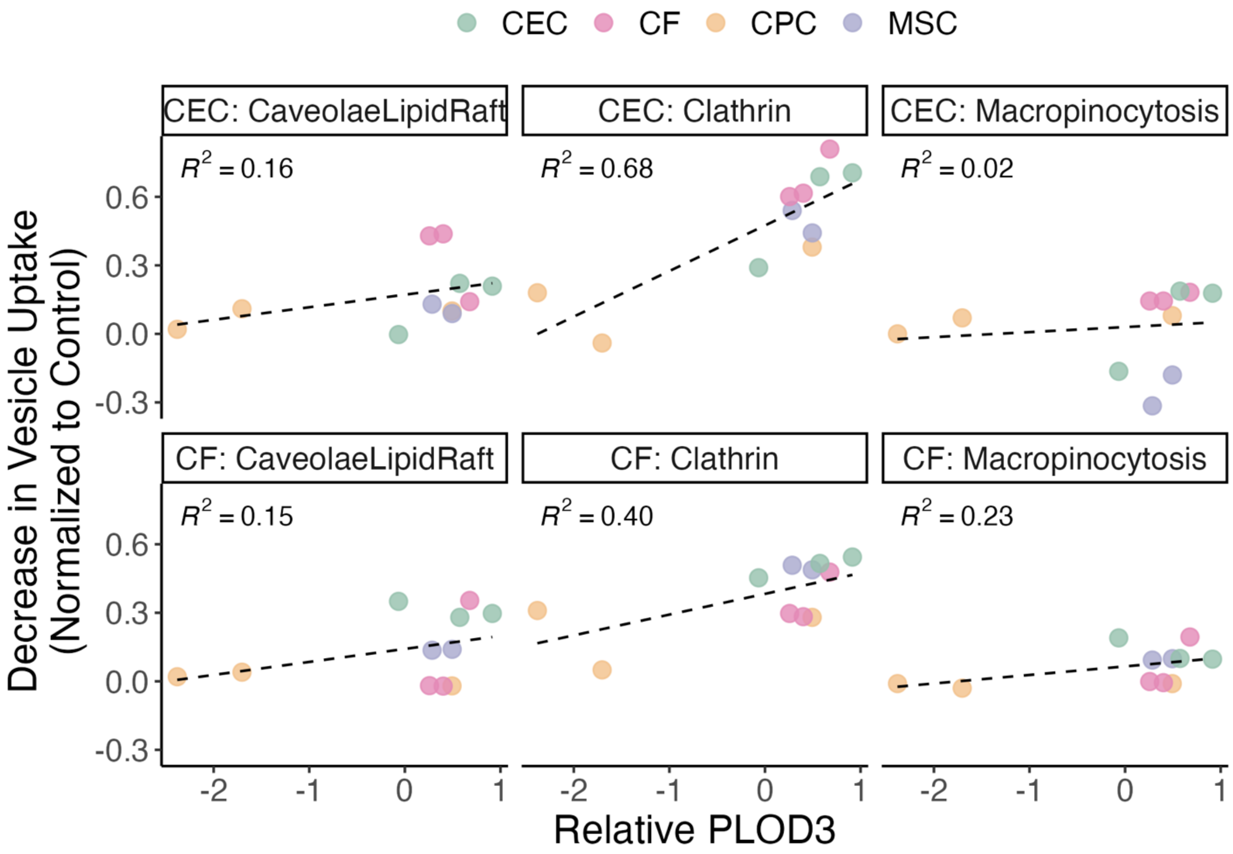

Supplementary Figure 22: Scatter plots of vesicle uptake by recipient cell type and uptake mechanism with increase in relative PLOD3.

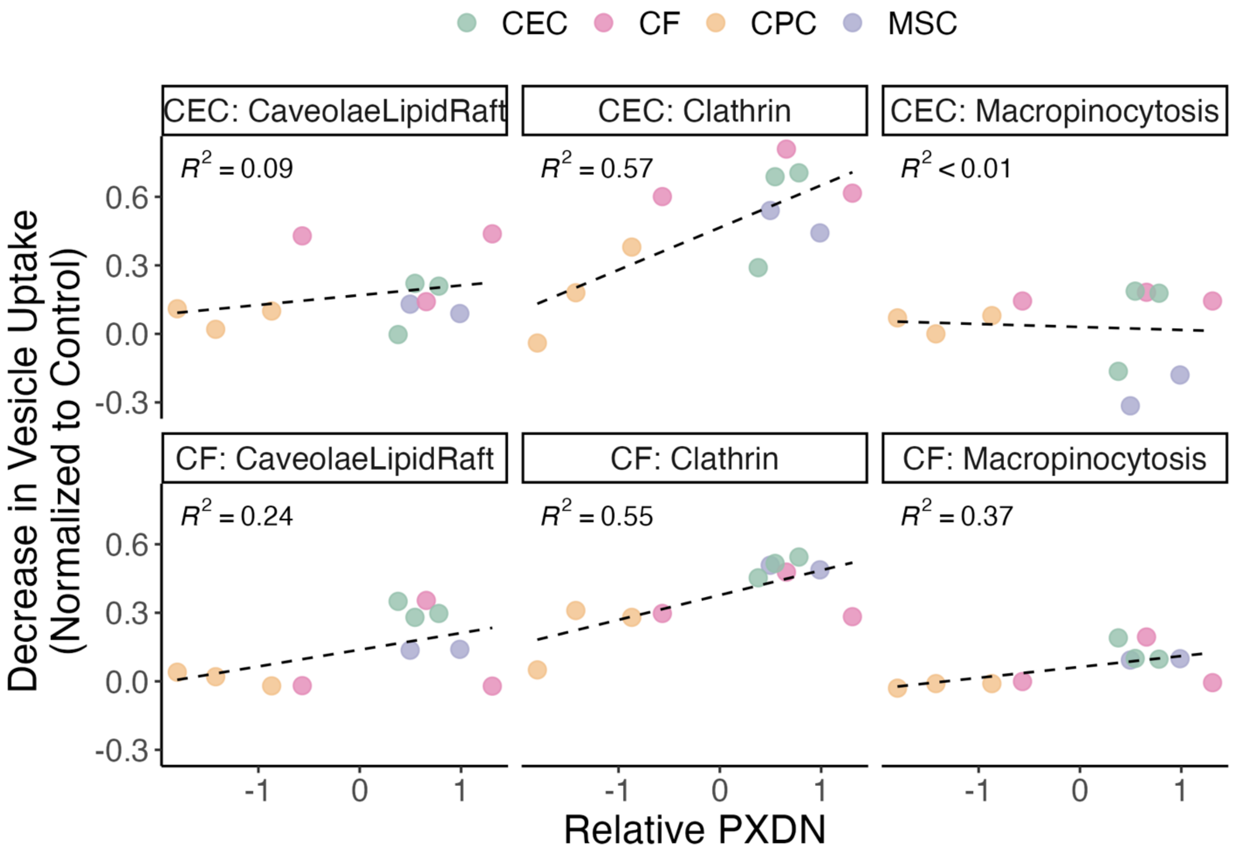

Supplementary Figure 23: Scatter plots of vesicle uptake by recipient cell type and uptake mechanism with increase in relative PXDN.

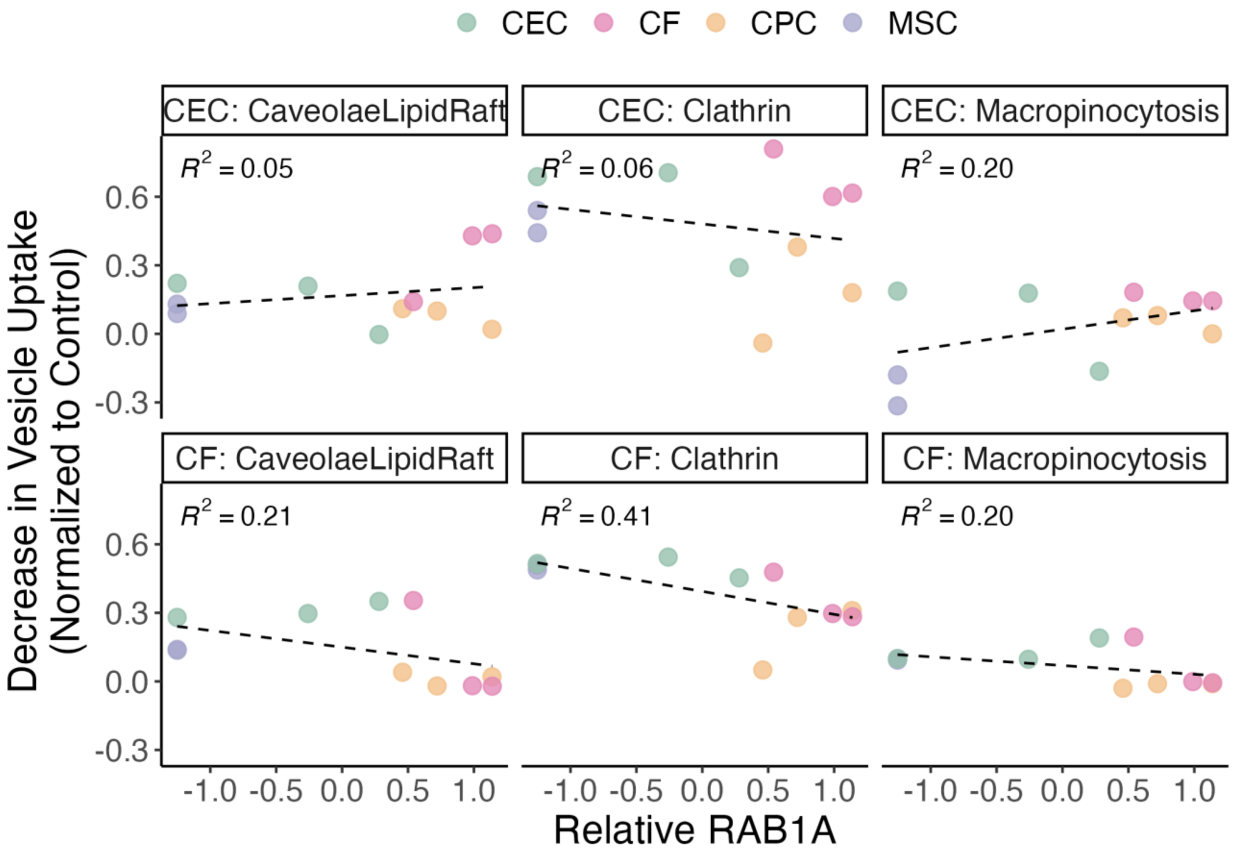

Supplementary Figure 24: Scatter plots of vesicle uptake by recipient cell type and uptake mechanism with increase in relative RAB1A.

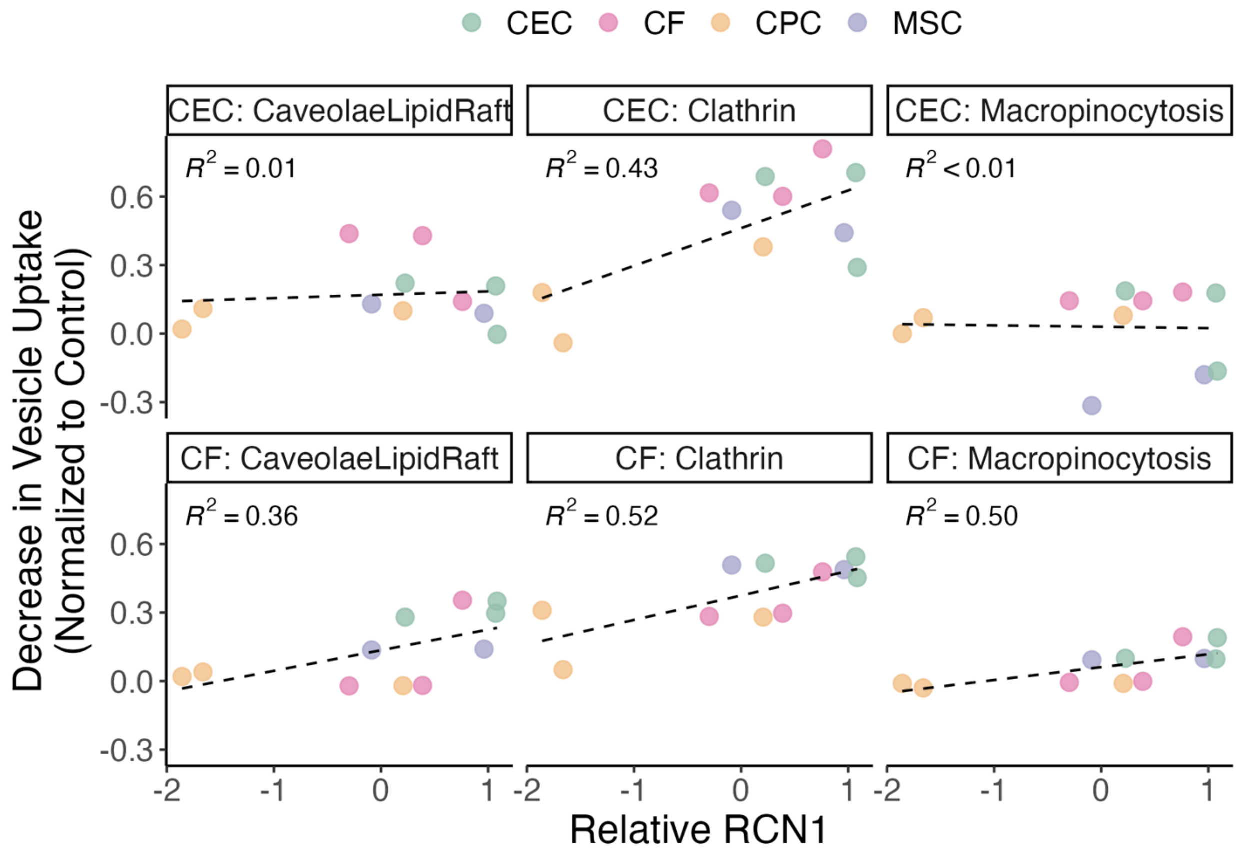

Supplementary Figure 25: Scatter plots of vesicle uptake by recipient cell type and uptake mechanism with increase in relative RCN1.

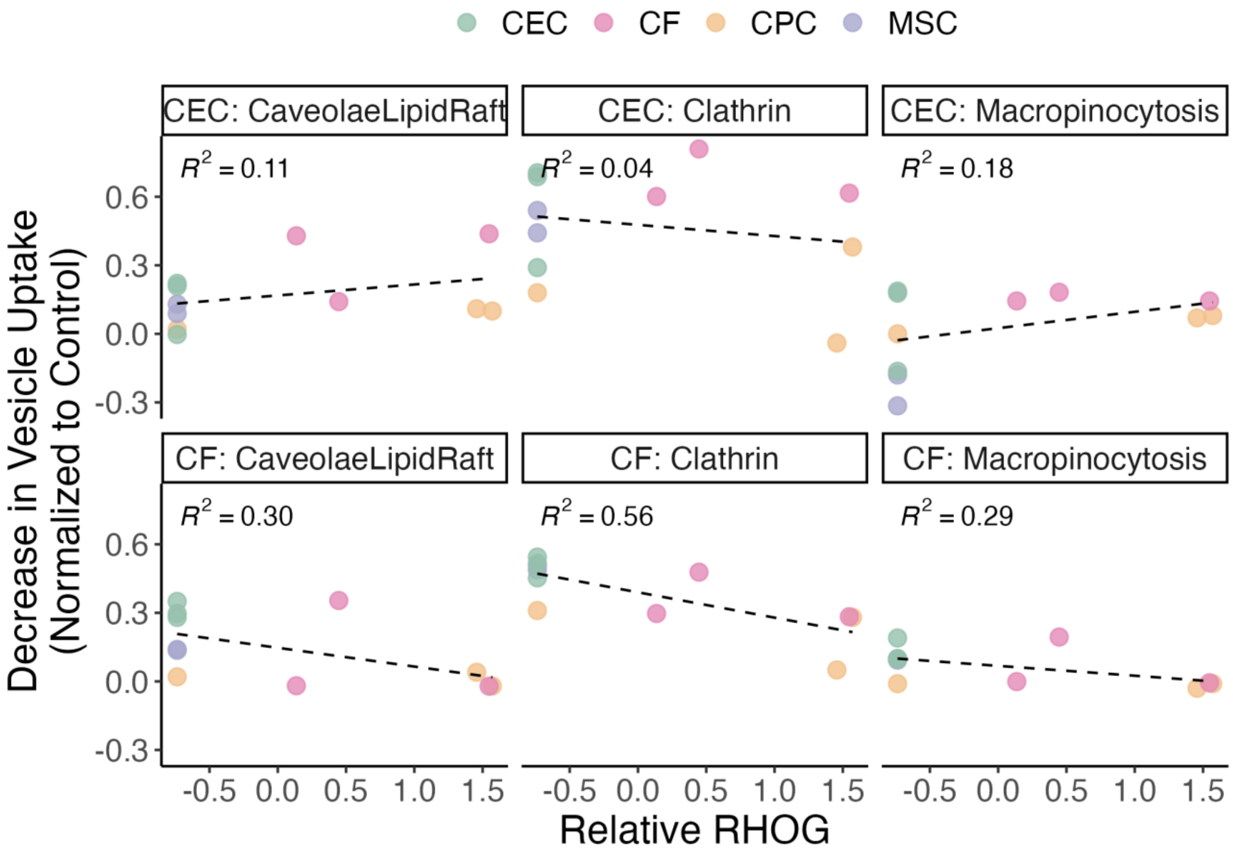

Supplementary Figure 26: Scatter plots of vesicle uptake by recipient cell type and uptake mechanism with increase in relative RHOG.

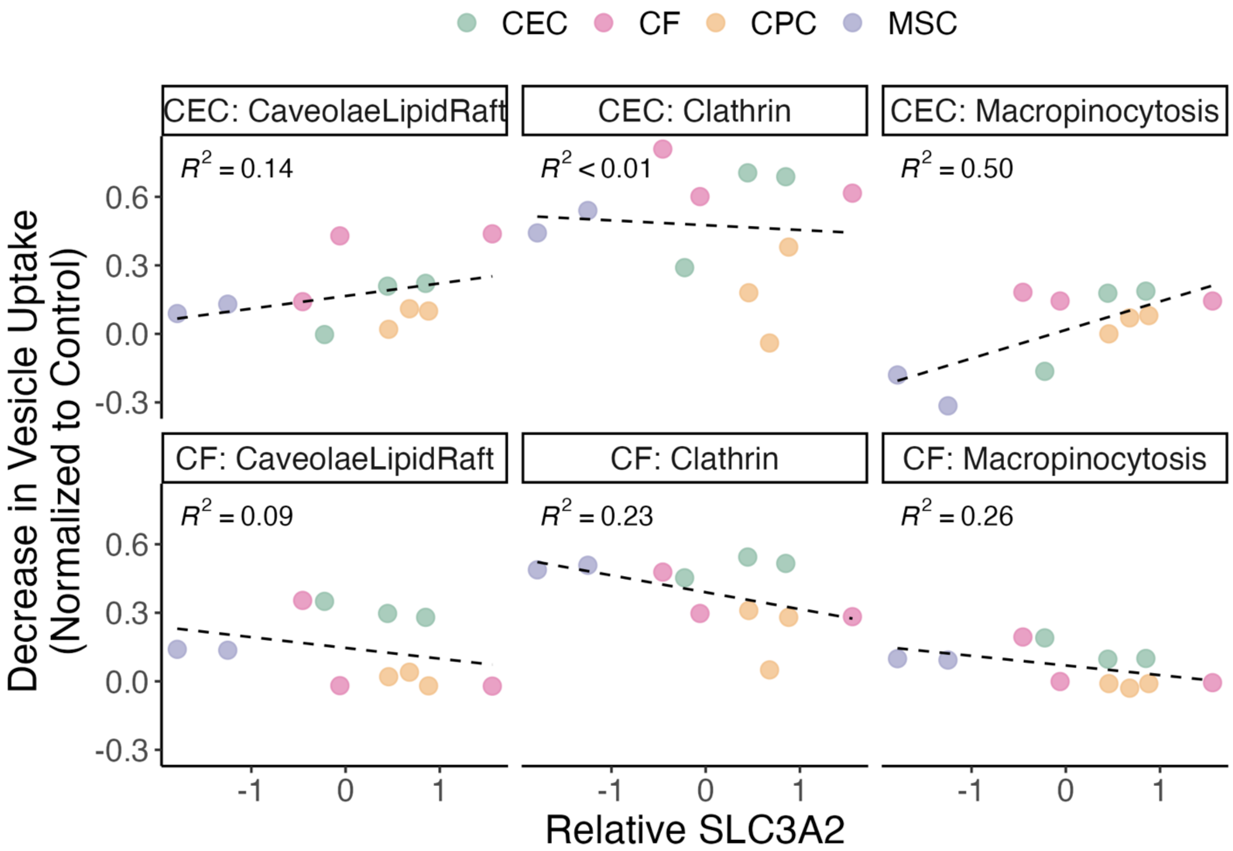

Supplementary Figure 27: Scatter plots of vesicle uptake by recipient cell type and uptake mechanism with increase in relative SLC3A2.

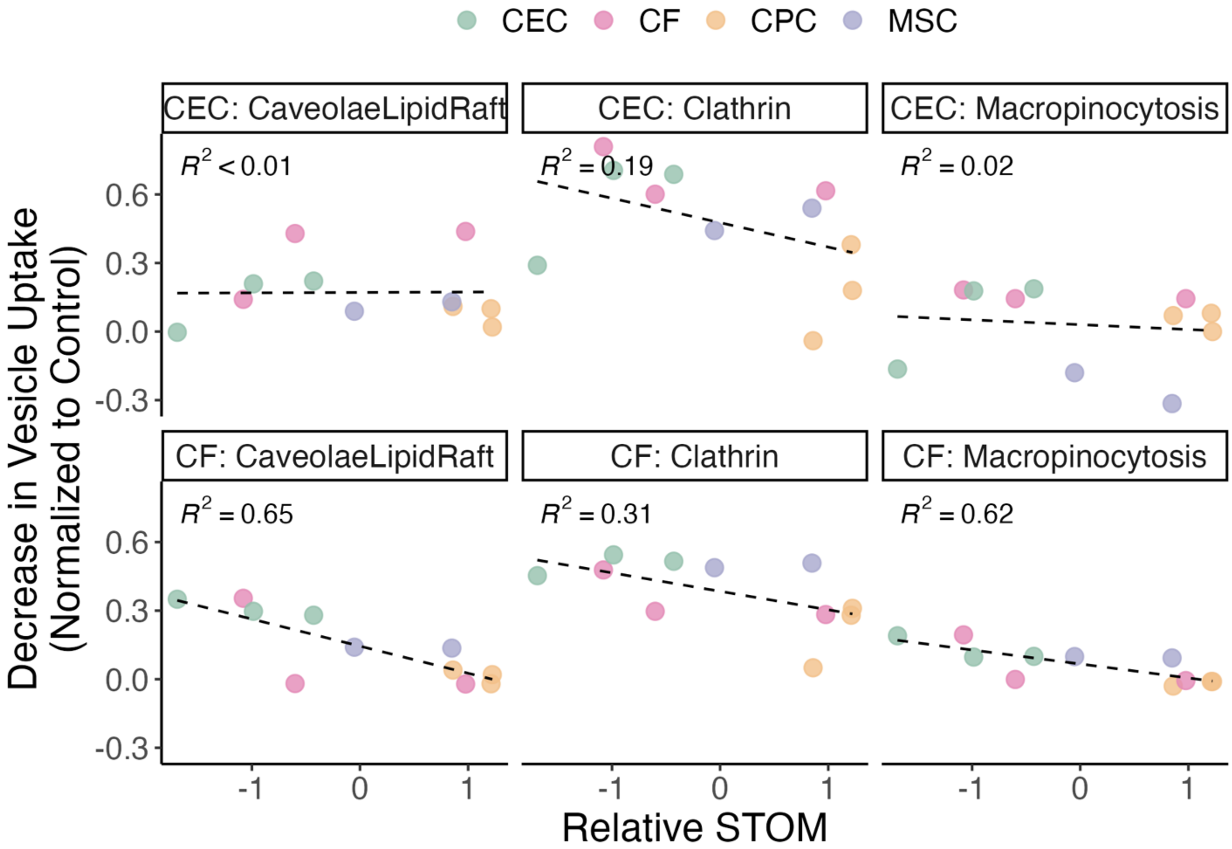

Supplementary Figure 28: Scatter plots of vesicle uptake by recipient cell type and uptake mechanism with increase in relative STOM.

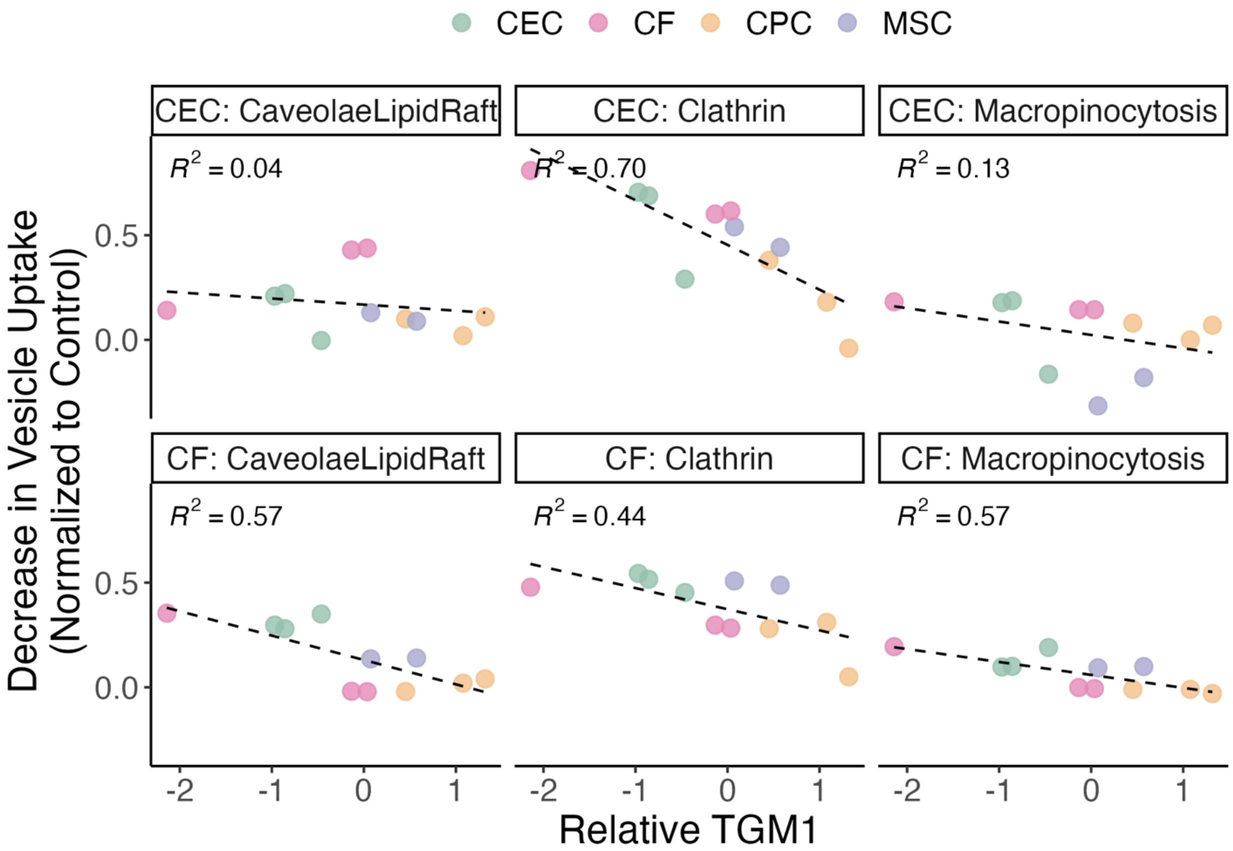

Supplementary Figure 29: Scatter plots of vesicle uptake by recipient cell type and uptake mechanism with increase in relative TGM1.

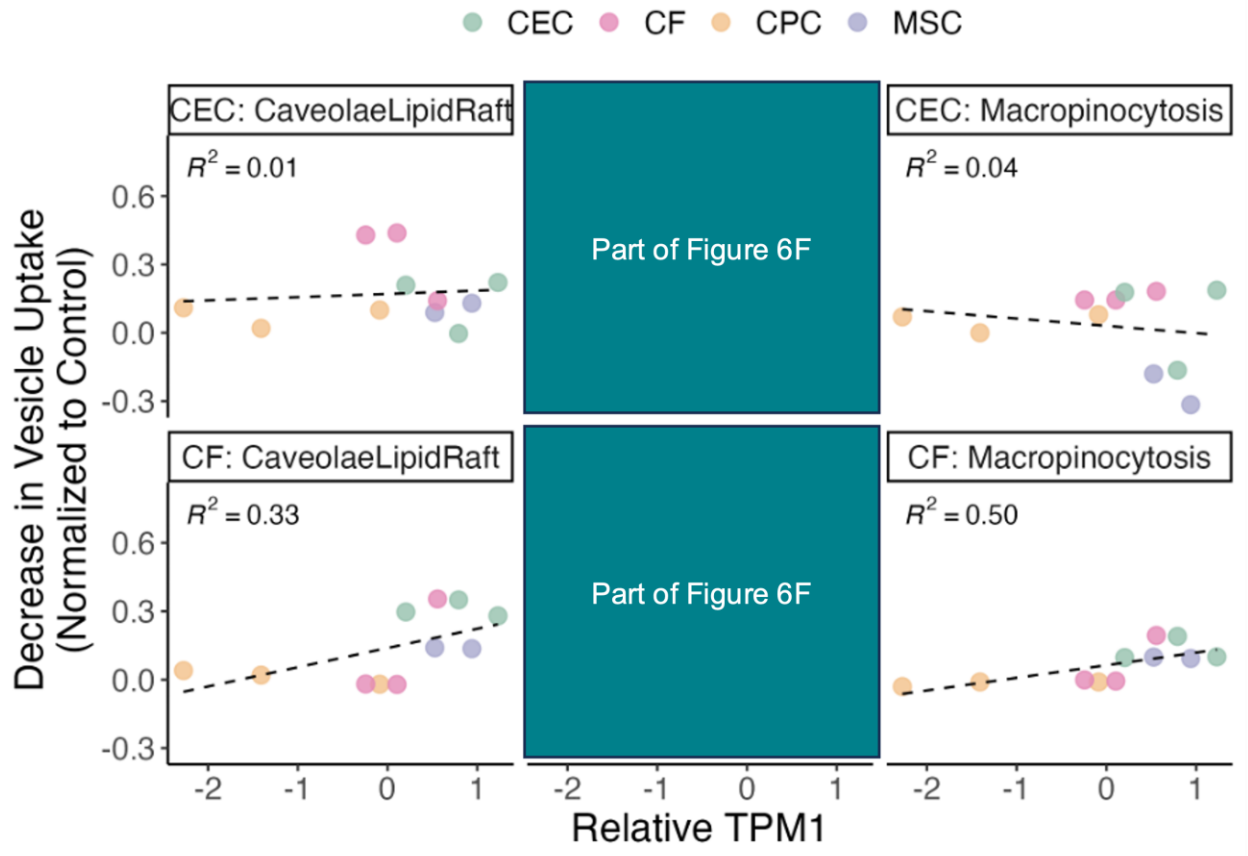

Supplementary Figure 30: Scatter plots of vesicle uptake by recipient cell type and uptake mechanism with increase in relative TPM1.

Supplementary Figure 31:

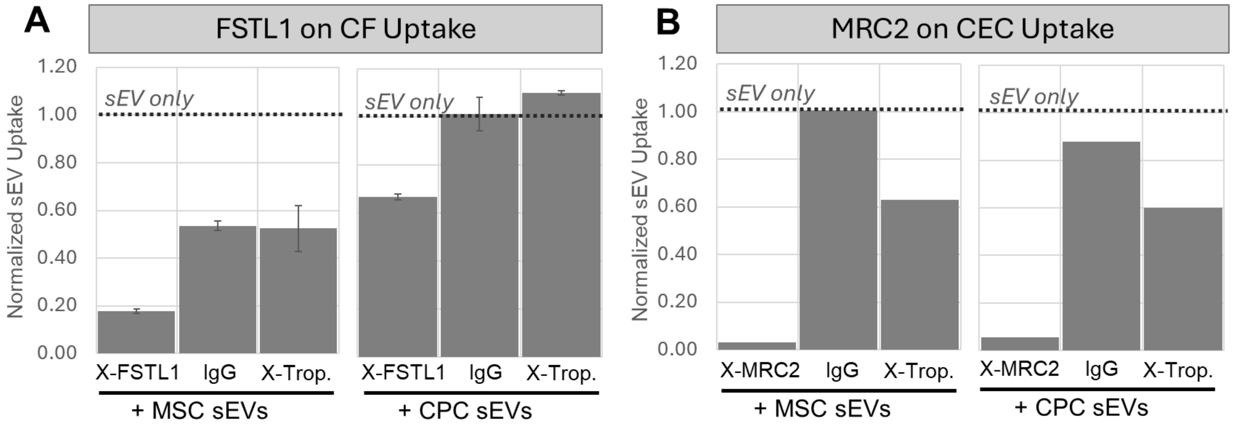

Supplementary Figure 31: Uptake of sEVs by CFs and CECs from MSC and CPC parent cell types assessed through flow cytometry after blocking FSTL1 or MRC2 proteins. (A) FSTL1 inhibition (X-FSTL1) reduces CF uptake of both MSC and CPC sEVs as predicted by the PLS model. (B) MRC2 inhibition (X-MRC2) reduced CEC uptake of both MSC and CPC sEVs as predicted by the PLS model. IgG and X-Trop. controls show limited impact of sEV uptake, confirming no steric effects during protein inhibition. X-Trop.=Troponin inhibition. Mean±S.D. n=1-2.

Supplementary Figure 32:

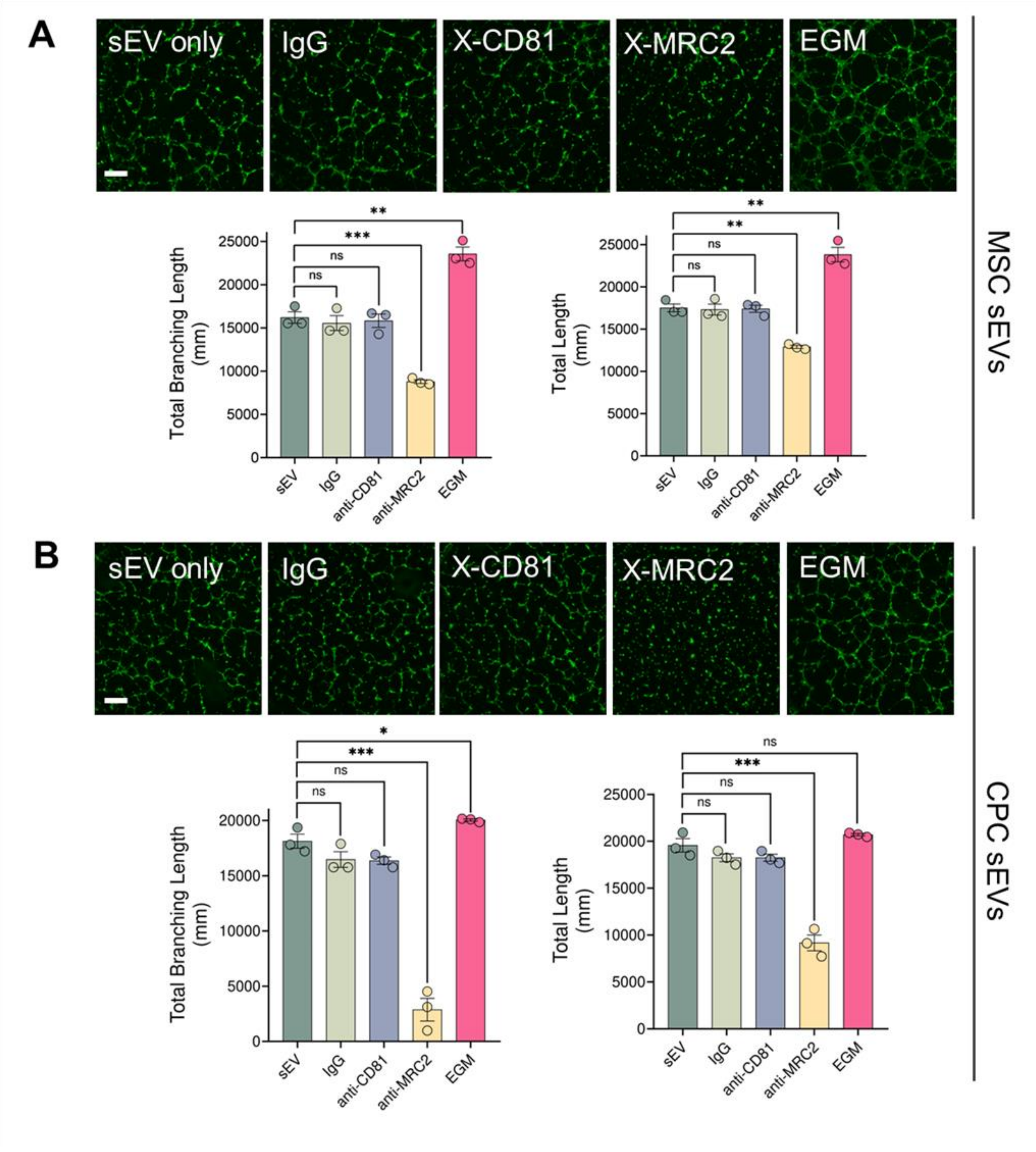

Supplementary Figure 32: Assessment of angiogenic response of CECs treated with MRC2-blocked sEVs through tube formation assay. (A) CECs treated with MSC-sEVs (B) CECs treated with CPC-sEVs. sEV group: serum-free CECs with sEV only (no inhibitors); negative control: IgG-treated sEVs and CD81-blocked sEVs; positive control: CECs in full-serum EGM media. Protein inhibition noted as anti-protein or X-protein. Mean ±SEM. Significance tested by two-sided Student's T-test. n.s.=not significant, *p<0.05, **p<0.01, ***p<0.001. Scale bar=200 μm.
